# Control System Design and Implementation of Battery-Assisted Quasi-Impedance-Source Inverter for Standalone Power Generation

**DOI:** 10.3390/s26185758

**Published:** 2026-09-10

**Authors:** Seyfettin Vadi, Meral Özarslan Yatak

**Affiliations:** Department of Electrical and Electronics Engineering, Faculty of Technology, Gazi University, 06560 Ankara, Turkey; ozarslanm@gazi.edu.tr

**Keywords:** battery-assisted qZSI, sinusoidal pulse width modulation, hybrid control, energy storage

## Abstract

**Highlights:**

**What are the main findings?**

- Low-output-voltage THD is demonstrated under both linear and nonlinear load conditions.
- DC-side battery/impedance network regulation and AC-side voltage control are achieved within a single-stage power-conversion structure.

**What is the implication of the main finding?**

- The proposed low-complexity PI+PR control framework provides a practical solution for coordinated DC- and AC-side regulation in standalone battery-assisted qZSI systems.

**Abstract:**

There is a growing need for high-efficiency power electronic converters that can effectively convert energy, regulate voltages, and enhance power quality in standalone power generators, as the use of renewable energy sources and battery energy storage devices increases. The quasi-impedance-source inverter (qZSI) has attracted significant interest due to its single-stage buck-boost operation, continuous input current, reduced reliance on passive elements, and increased reliability. In this paper, the control strategy and implementation of the qZSI with battery assistance for standalone photovoltaic energy generation are discussed. To analyze the operational characteristics and design the control strategy of the qZSI, the system equations are linearized around the nominal operating point to develop a small-signal model, from which the direct current (DC) side and alternative current (AC) side transfer functions are derived and used as the basis for controller design. Using the proposed model, hybrid controllers are designed to control the shoot-through duty cycle, maintain DC link voltage stability, and battery charging to achieve stable power generation. Furthermore, the SPWM technique is applied to produce AC power with minimal harmonic content and higher efficiency. Application results show stable dynamic behavior, effective battery energy management, improved voltage regulation, and reduced harmonic distortion in the output waveform. The main contribution is a low-complexity coordinated PI and PR control framework for standalone battery-assisted qZSI operation, experimentally validated under DC- and AC-side disturbances without requiring an additional battery-side power-conversion stage.

## 1. Introduction

Power converters play a crucial role in solar energy generation systems, as they convert energy into a suitable form for electrical devices or the grid. The basic impedance-source inverters (ZSIs) and their advantages are presented in [1]. An impedance-source inverter (ZSI) does not require an additional boosting device, thereby reducing system cost, complexity, and losses. Different configurations of ZSI topologies have been presented and reviewed in the recent literature studies [2]. Due to their advantages, impedance-source inverters have been the subject of extensive research; therefore, this converter has also been investigated for on-grid systems [3,4,5]. A battery is also required to maintain the input voltage [6,7]. To improve ZSI, a type of inverter called a qZSI has recently been investigated. The qZSI has many advantages, especially its suitability for distributed energy generation applications. It is a topology that draws a constant current from the input and allows for operation over a wide input-voltage range. In the literature, control strategies and grid-connected systems for qZSI are analyzed [8,9]. The literature indicates that studies have been conducted across various fields, including motor drive applications, battery charging systems, and renewable energy sources. Due to qZSIs’ single-stage capability to both boost the input voltage and convert DC to AC, they are widely used in solar energy systems [10,11]. qZSIs can also be used as step-switched capacitor DC–DC converters [12] and DC–AC converters [13]. Many PWM techniques are discussed in [14,15,16,17] to boost the input voltage. Different control strategies, such as sliding-mode control (SMC), are also used for DC-side voltage regulation [18,19,20]. SMC is used to improve the dynamic response of capacitor-voltage control in an on-grid qZSI [21]. The advantage of the SMC over the PI controller has been demonstrated by its ability to reduce ripples in the reference capacitor voltage. In [22], a new short-circuit-based PWM technique for discrete control of ZSI is presented.

In addition to conventional PI, PR, and sliding-mode control strategies, recent studies have increasingly focused on adaptive, resilient, fuzzy, and event-triggered control approaches to improve robustness against model uncertainties, external disturbances, communication constraints, and cyber-physical disruptions. Hu et al. developed a resilient distributed fuzzy load frequency control strategy for multi-area power systems subject to cross-layer random denial-of-service attacks. Their approach employs an interval type-2 Takagi–Sugeno fuzzy framework and demonstrates improved frequency regulation and robustness under uncertain system parameters and cyber-physical disturbances [23]. Event-triggered adaptive control has also received considerable attention because of its ability to balance tracking performance and communication/computational requirements. For example, adaptive event-triggered fuzzy tracking controllers have been developed for uncertain nonlinear systems to reduce communication and computational burden while maintaining tracking performance [24,25,26,27]. More recently, Hu and Ma proposed an adaptive event-triggered tracking control method based on switching functions to compensate for unmodeled dynamics and external disturbances, and to guarantee finite-time tracking performance [24]. These studies demonstrate the effectiveness of advanced adaptive control mechanisms in improving robustness and tracking performance. However, most of these approaches are primarily intended for networked, multi-agent, or general nonlinear systems and require adaptive laws, fuzzy approximators, event-triggering mechanisms, switching functions, or additional computational resources. Their control objectives therefore differ from converter-level regulation in standalone battery-assisted qZSI systems, where coordinated DC-side energy regulation and AC-side sinusoidal voltage control must be achieved with a practically implementable control architecture.

Despite these advances in adaptive and intelligent control, there remains a need for a simple, computationally efficient, and experimentally validated control architecture specifically tailored to standalone battery-assisted qZSI systems. Existing advanced adaptive and event-triggered methods mainly address uncertainties, communication constraints, or distributed regulation in networked and nonlinear systems, whereas the converter-level problem considered here requires simultaneous regulation of the battery-assisted DC-side dynamics and the sinusoidal AC output voltage. Moreover, the additional computational complexity associated with adaptive laws, fuzzy approximators, and event-triggering mechanisms may not always be desirable for real-time implementation in power electronic converters. To address this gap, this study develops a coordinated hybrid PI+PR control architecture in which the PI controller regulates the DC-side variables of the battery-assisted impedance network, while the PR controller provides sinusoidal AC-side voltage regulation. The proposed approach emphasizes practical implementation with a reduced number of sensing elements and is experimentally evaluated under variations in input voltage, battery current, linear and nonlinear loads, and reference operating conditions. Therefore, the main contribution of this work lies in achieving coordinated DC- and AC-side regulation with relatively low control complexity while demonstrating its feasibility through experimental implementation [24,25,26,27].

This paper presents a standalone qZSI system with hybrid control for both the AC and DC sides. Furthermore, a configuration for battery charging in microgrid applications is proposed, allowing the higher voltage battery to be charged with an appropriate charging current, and utilizing the voltage across the capacitor to charge the battery connected to it. The system’s dynamic performance is analyzed under various conditions, including variations in input, battery charging current, and AC load. Experimental and hardware results confirm the effectiveness of the proposed control structure for all the above cases. This work aims to present an efficient method for photovoltaic power generation systems with energy storage, operating in parallel with a single-stage DC–DC boost converter and a DC–AC converter, and to control and optimize the control parameters. For this purpose, a PI- and PR-based hybrid controller is designed, and it is proposed to develop a robust dynamic response and low-cost hybrid control technique to ensure system stability under varying load conditions, reference voltage variations, and different output voltages, and to minimize the current battery ripple without using additional hardware structure. Accordingly, the PI controller is preferred for DC-side voltage stability, and the PR controller is preferred for AC-side voltage stability. Thus, qZSI control is achieved using a PI+PR hybrid control method. The performance of the proposed control method is evaluated using the same input, output voltages and reference voltages. Additionally, the proposed controller is tested experimentally in the application setup.

The main contribution of this study is the development and experimental validation of a low-complexity coordinated DC–AC control framework for a standalone battery-assisted qZSI. Rather than introducing an additional power-conversion stage or employing computationally intensive adaptive or nonlinear control algorithms, the proposed approach integrates DC-side PI regulation and AC-side PR regulation within the inherent single-stage buck-boost inversion capability of the qZSI.

More specifically, the main contributions of this work can be summarized as follows:

- A coordinated PI+PR control architecture is developed for standalone battery-assisted qZSI operation, where the DC-side controller regulates the battery/impedance network dynamics through the shoot-through duty ratio, while the AC-side PR controller maintains sinusoidal output-voltage regulation.
- The controller design is supported by a small-signal model that considers the dynamic interaction between the DC- and AC-side variables. The direct and cross-coupling characteristics are analyzed to clarify the applicability of the decentralized PI+PR control structure.
- A systematic controller-parameter selection and sensitivity analysis is introduced to quantify the effects of PI and PR gain variations on settling time, overshoot, and steady-state tracking error. Closed loop pole and stability analyses are additionally employed to verify local stability around the investigated operating points.
- The proposed control strategy is implemented on a 1 kW laboratory prototype and experimentally evaluated under load variations, battery-current variations, input voltage disturbances, and linear and nonlinear loading conditions. The experimental results provide quantitative assessment in terms of transient performance and harmonic distortion.

Therefore, the novelty of the proposed approach lies not merely in combining PI and PR controllers, but in providing a practically implementable and experimentally validated coordinated control framework for standalone battery-assisted qZSI operation, supported by systematic controller design, stability assessment, cross-coupling analysis, and quantitative dynamic performance evaluation.

## 2. Battery-Assisted qZSI Topology

**Figure 1:**
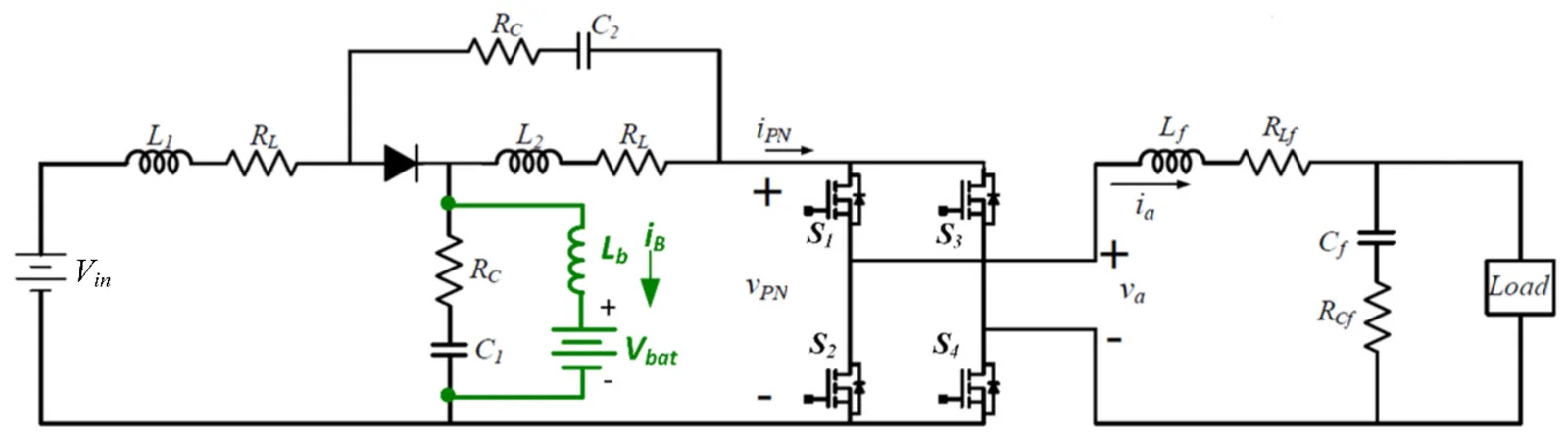
Battery-assisted qZSI topology.

The proposed battery-assisted qZSI topology is given in Figure 1. The battery bank is connected in parallel to the impedance network, which is connected to a single-phase H-bridge inverter. The qZSI impedance network comprises coils (*L*_1_, *L*_2_), capacitors (*C*_1_, *C*_2_), and diodes. The battery bank is connected in parallel to capacitor *C*_1_. The system is designed so that the DC-side control regulates the battery charging current. The AC-side control regulates the grid supply current via shoot-through control, regardless of the battery’s state of charge (SOC).

In general, if we specify the qZSI’s operation for an input voltage *V_in_*, it always operates in two states. One is the non-shoot-through mode, and the other is the shoot-through mode. When the inverter operates in short-circuit mode, the two switches on a single arm conduct simultaneously, shorting the impedance circuit’s output, and the diode enters reverse operation. That is, the switching duty ratio (*D*) controls the stepping of the input voltage.

**Figure 2:**
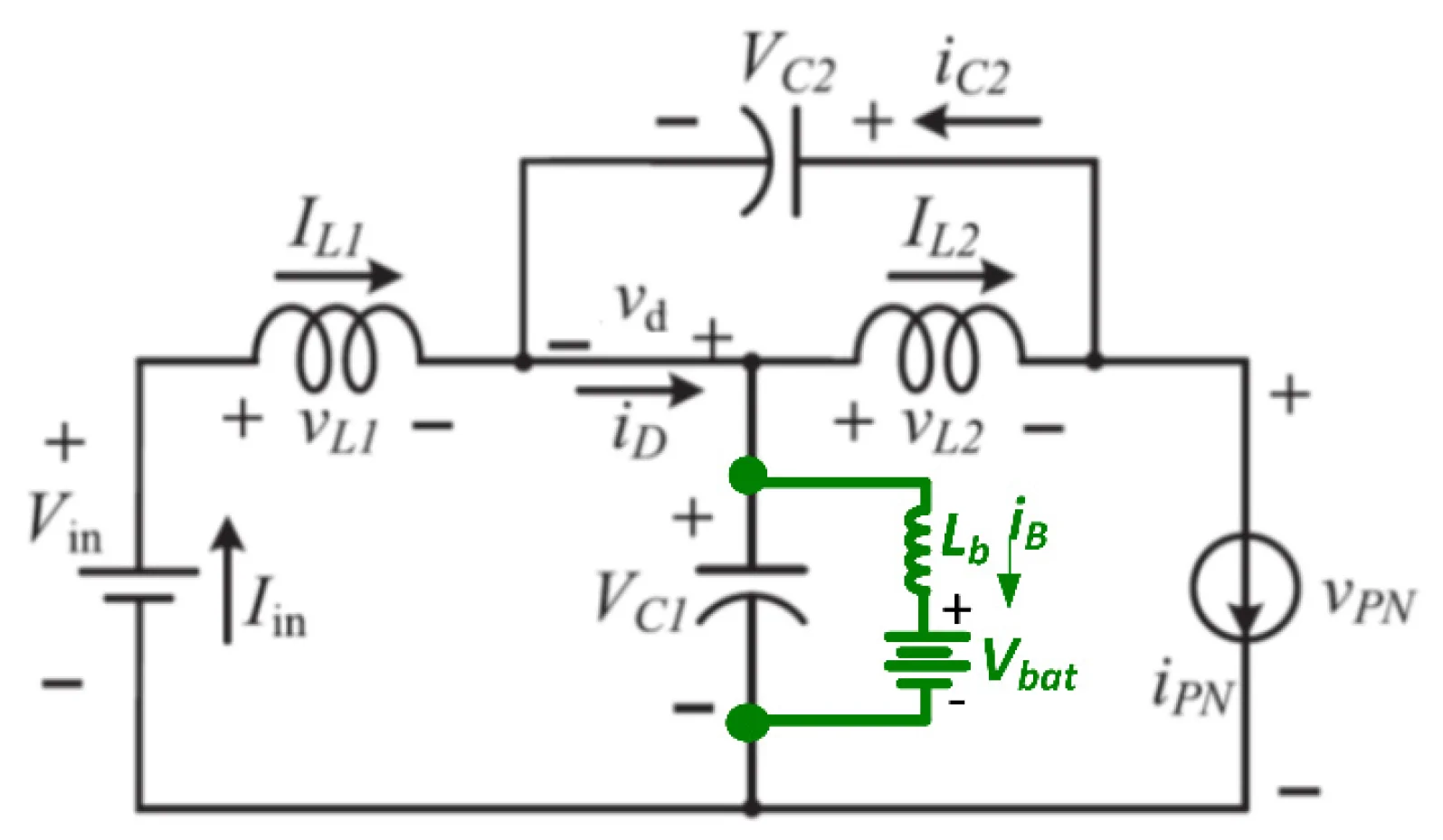
Non-shoot-through statement.

In the non-shoot-through operating state of the inverter, it operates as a conventional voltage-source inverter (VSI), in which two switches in an inverter arm do not operate simultaneously. Therefore, the total time period (*T*) will be the sum of the short-circuit operating state time (*T*_0_) and the non-short-circuit operating state time (*T* − *T*_0_). *D* can be expressed as *D* = *T*_0_/*T*. For a symmetrical qZSI impedance network with identical inductances *L*_1_ and *L*_2_ and identical capacitances *C*_1_ and *C*_2_, the mathematical analysis of the qZSI is given below [8]. As shown in Figure 2, the system equations for the non-short-circuit mode operation can be written as follows:

(1) VL1=Vin−VC1VL2=−VC2VB=VC1−VLBiB=1LB∫0tVLBdt

(2) $$V_{DC}=V_{C1}-V_{L2}=V_{C1}+V_{C2},\;V_{D}=0$$

**Figure 3:**
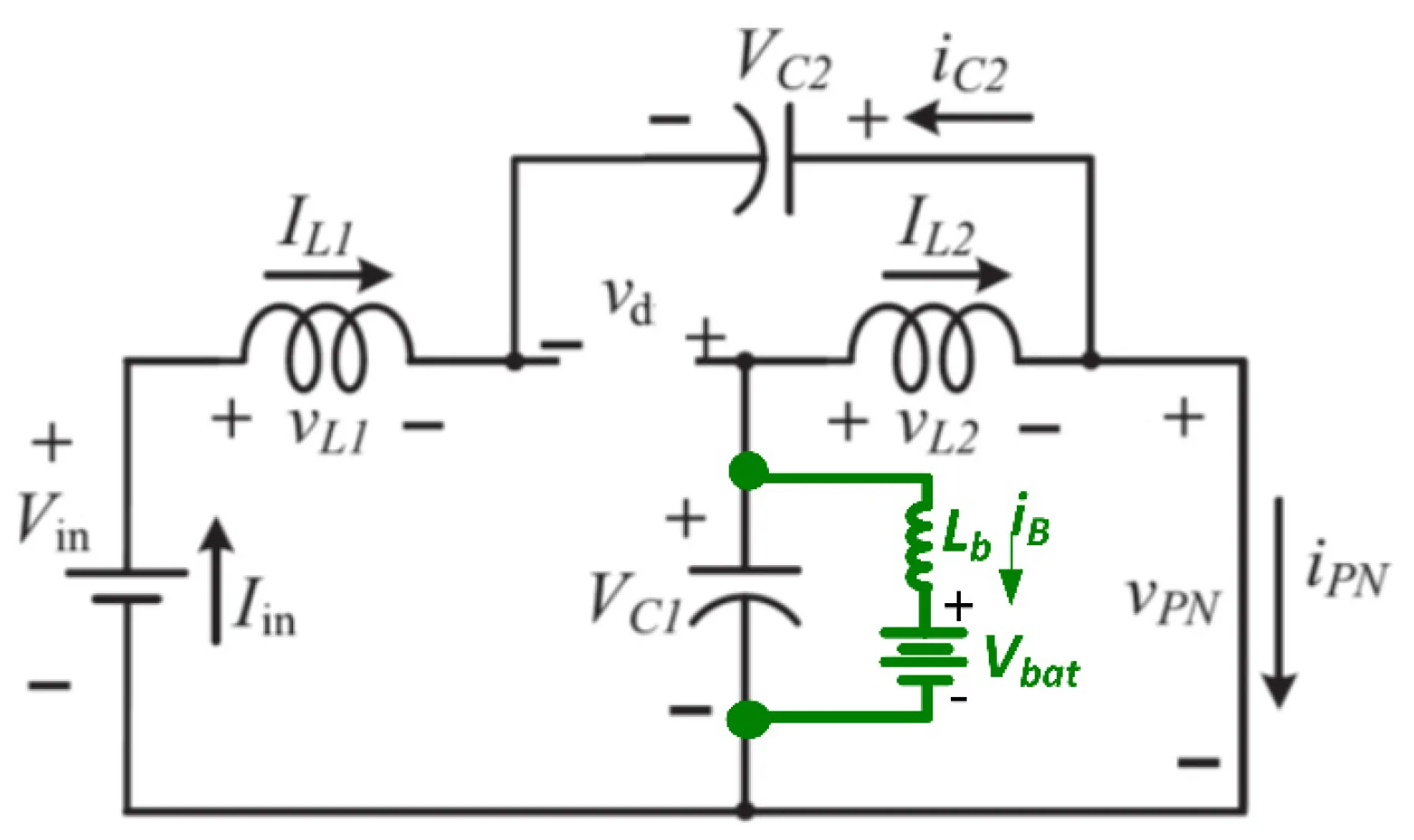
Shoot-through statement.

The system equations are shown in Figure 3. The equations for the short-circuit mode (shoot-through) can be written as in Equations (3) and (4):

(3) VL1=VC2+VinVL2=VC1VB=VC1−VLBiB=1LB∫0tVLBdt

(4) $$V_{DC}=0,V_{D}=V_{C1}+V_{C2},V_{D}=V_{BAT}+V_{C2}$$

The average voltage across both coils in a steady state is zero during one switching cycle. From Equations (1) and (3),

(5) $$V_{L1}=\frac{T_{0}\left(V_{C2}+V_{in}\right)+(T-T_{0})(V_{in}-V_{BAT})}{T}=0$$

(6) $$V_{L2}=\frac{T_{0}\left(V_{C1}\right)+(T-T_{0})({-V}_{C2})}{T}=0$$

By solving Equations (5) and (6),

(7) $$V_{C1}=\frac{T-T_{0}}{T-2T_{0}}V_{in}$$

The DC bus voltage can be given as in Equations (8)–(10):

(8) $$V_{DC}=V_{C1}+V_{C2}=\frac{T}{T-2T_{0}}V_{in}$$

(9) $$V_{DC}=\frac{1}{1-2D}V_{in}$$

(10) $$V_{DC}=BV_{in}$$

*B* is the boost factor of the qZSI. Equations (1)–(10) show the mathematical expression of the proposed topology. Here, the voltage across the capacitor charges the batteries at current *i_B_* in both shoot-through and non-shoot-through modes.

## 3. Design of the Hybrid Control Method

The derivation of the transfer functions required for the design of the hybrid controller involves linearization of the battery-assisted qZSI about its operating point. The equilibrium conditions, based on the steady-state relationships developed in Section 2 (Equations (1)–(10)), form the basis of this linearization process, in which small signals about the operating point are assumed to generate linearized models of the DC- and AC-sides. The linearized model on the DC side links changes in the shoot-through duty ratio to changes in the capacitor voltage and battery current, and hence serves as the basis of the design of the PI controller. On the AC side, the linearized model yields the transfer function from the modulation signal to the output voltage, which forms the basis for the PR controller design.

**Figure 4:**
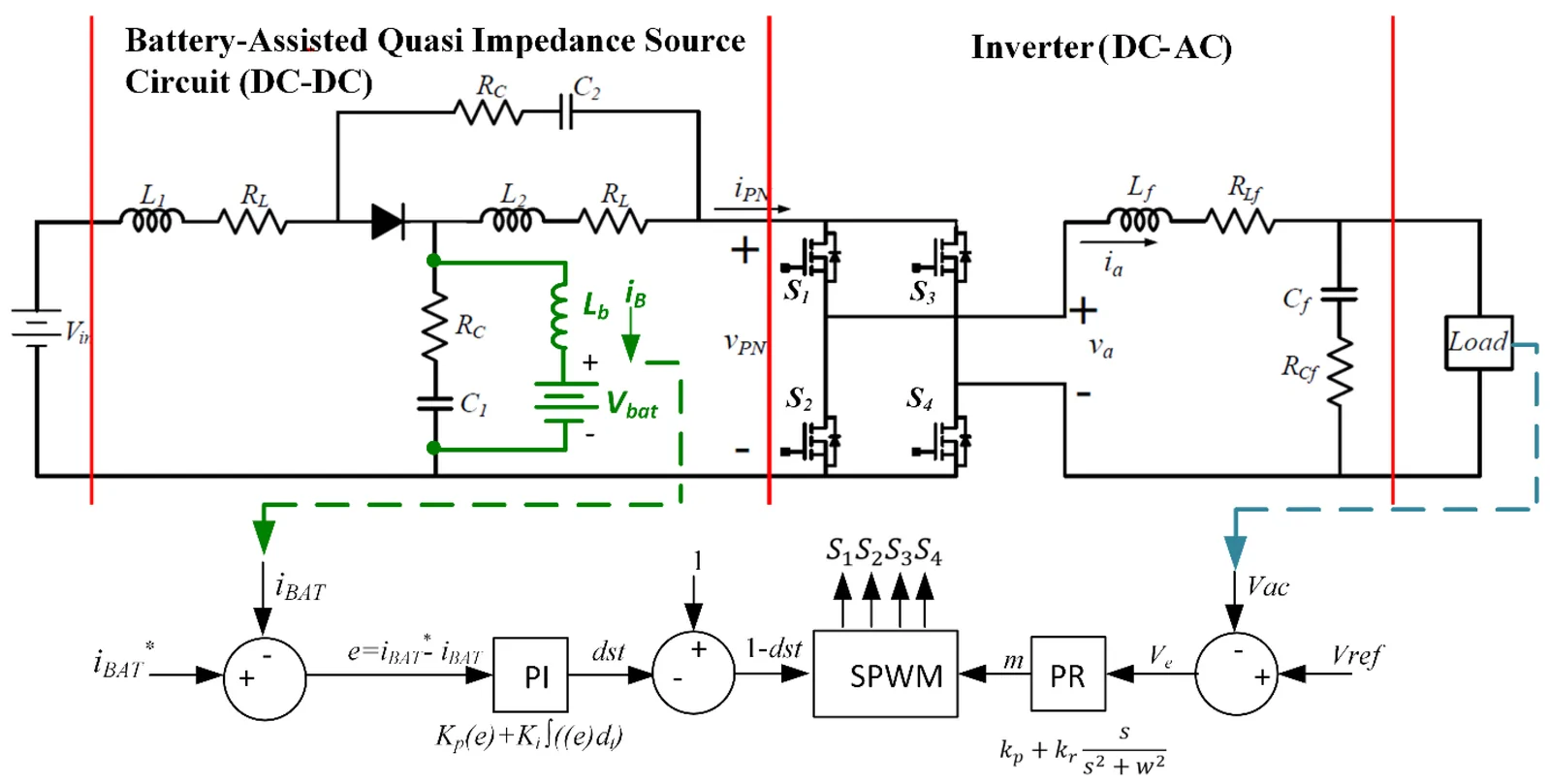
Block scheme of the proposed standalone battery-assisted qZSI.

The overall block schema of the proposed system with the hybrid control method is shown in Figure 4. Since the inverter output is the grid in standalone mode, the purpose of the AC-side control is to regulate the inverter supply current according to the current reference under any disturbance in the DC input voltage. The objective of the DC-side control is to regulate the battery charging current in constant current mode and regulate the voltage in constant voltage mode depending on the SOC level of the battery. The minimum battery voltage for power flow to the load must be high enough to maintain the inverter DC link voltage above the required minimum. For the AC side, closed-loop current control will be preferred. According to Figure 4, the amplitude signal for controlling the AC side is derived using the PR control method. This amplitude signal is multiplied by the constant voltage reference to obtain the real AC voltage reference signal. The reference AC voltage is compared with the inverter output voltage, and the resulting error signal is applied to the PR controller, which generates the modulation signal.

On the DC side, since the battery is connected via capacitor *C*_1_, the current through the battery must be regulated in constant current mode, and the capacitor voltage must be maintained at a constant voltage. In constant current mode, the actual current is compared with the reference value, and the error generated is fed to the PI controller. The PI controller generates a switching signal to boost the input voltage. The digital controller logically combines the modulation and switching signals using a short-circuit mode-based SPWM technique [22] and feeds them to the master switching controller. This modulation method provides a sinusoidal, symmetrical distribution of short-circuit mode states, resulting in an improved current profile and enhanced discrete control capability for modulation-index control. The LC filter connected to the AC-side output suppresses harmonics. The system and control parameters are given in Table 1.

### 3.1. DC-Side Control of Battery-Assisted qZSI

The proposed control strategy is structured to consider both the DC and AC sides of the qZSI. The control method can be defined as regulating the capacitor voltage (*V_C_*_1_). The first control part of the topology is the DC side, commonly referred to as the step-up converter. The DC side is the impedance circuit of the topology and consists of two coils, two capacitors, and a diode. The control variables on the DC side are *I_L_*_1_, *I_BAT_*, *I_L_*_2_, *V_C_*_1_, and *V_C_*_2_.

The DC bus voltage (*V_PN_*) is equal to the sum of the capacitor voltages *V_C_*_1_ and *V_C_*_2_. Both the DC and AC sides of the battery-assisted qZSI are controlled. On the DC side, one or both of the bus voltages *V_C_*_1_, *I_L_*, or *V_PN_* are used together to speed up the process. In this study, the battery current, *I_BAT_*, is selected as the control parameter for the DC side, and a PI controller is applied to the battery-assisted qZSI. The error signal (*e_BAT_*), *I_BAT_*, and its reference are compared as in Equation (11).

(11) $$e_{BAT}={I_{BAT}}^{*}-I_{BAT}$$

The error parameter is used in a PI control method, as in Equation (12).

(12) $${d_{st}=K_{P}e}_{BAT}+K_{i}\int e_{BAT}dt$$

**Figure 5:**
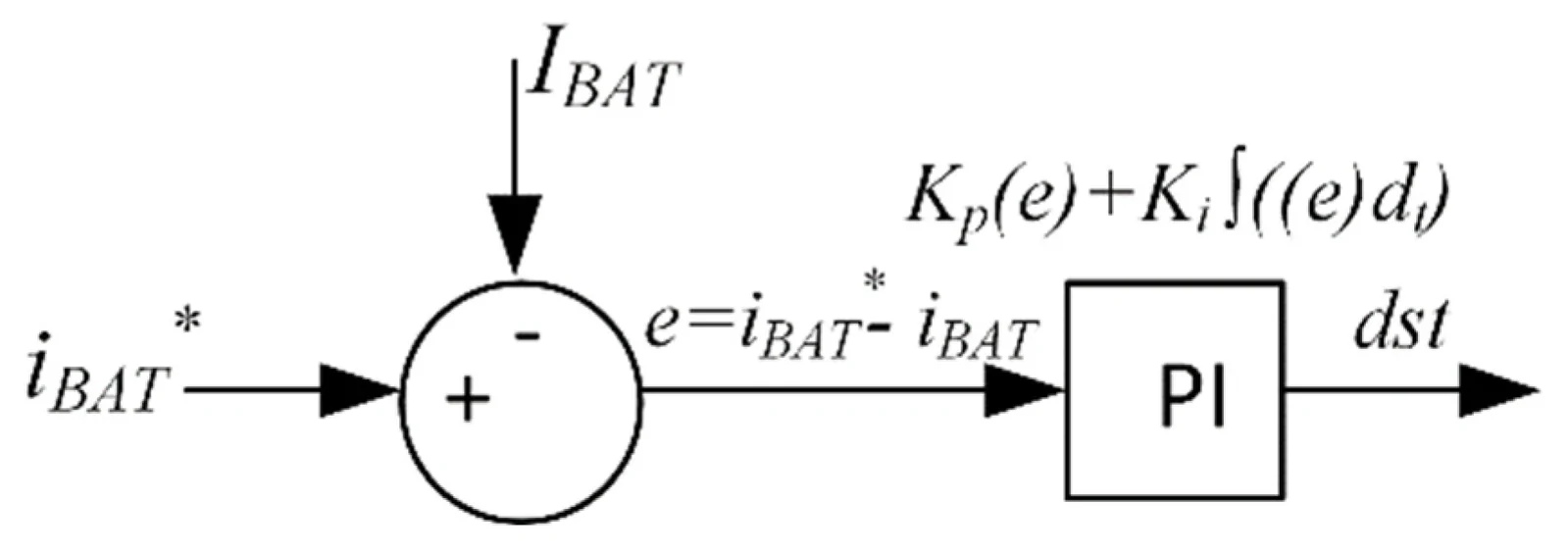
Block scheme of Equation (12).

The short-circuit mode duty ratio (*d_st_*) is determined for bus voltage step-up on the DC side of the inverter. The block scheme of the equation for this ratio is given in Figure 5. The parameters *K_p_* and *K_i_* are proportional and integral constants, respectively. These parameters are used to minimize the DC-side voltage tracking error.

The reference voltage *V*_*C*1_ and the shooting-through duty ratio are calculated by using the qZSI steady-state equations (Equations (7)–(9)). These determine the linearization point for the small-signal analysis and also the set-point for the PI controller.

### 3.2. AC-Side Control of Battery-Assisted qZSI

The other control component of the topology is the inverter. In this part, the control variable is the output voltage (*v_ac_*). The PR control method aims to regulate the output voltage. *v_ac___ref_* is the reference of *v_ac_* and can be obtained as in Equation (13).

(13) $$V_{e}=v_{ac\_ref}-v_{ac}$$

**Figure 6:**
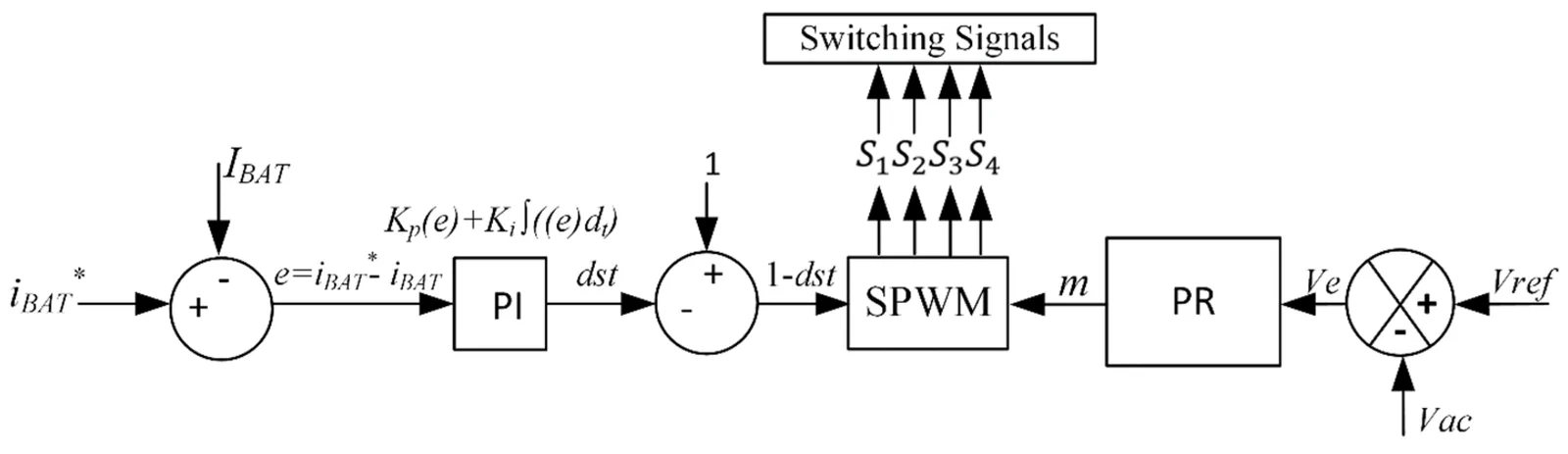
Block scheme of the PI+PR control of the battery-assisted qZSI.

The error (*V_e_*) parameter is applied to a PR control method. The controller eliminates the steady-state error for the AC side of the battery-assisted qZSI and generates the modulation signal. As a result, the PI controller generates the switching signals for the DC side, and the PR controller generates them for the AC side. Figure 6 shows the PWM switching signal generation scheme for a single-phase battery-assisted qZSI. The parameters *K_p_* and *K_r_* (proportionality and resonance constants, respectively) of the PR controller are determined using the trial-and-error method. The AC-side voltage tracking error is shown in Figure 6.

The error due to the difference between the measured AC voltage and the reference output voltage is controlled using a PR control method, thereby ensuring system stability.

The requirement for zero steady-state error at 50 Hz, established by the AC-side voltage equation (Equation (13)), motivates the use of the PR controller structure in Equation (14), whose parameters are subsequently tuned using the frequency domain analysis described in Section 4. However, it is important to note that the small-signal model presented in this paper treats the DC and AC control loops as separate, independent subsystems. Any cross-coupling effect between the battery current on the DC side and the output voltage on the AC side is not considered. Although the experimental results have validated the model’s performance, it may be necessary to consider the effect of coupling in further studies.

## 4. Parameter Design of the Hybrid Control Method

**Figure 7:**
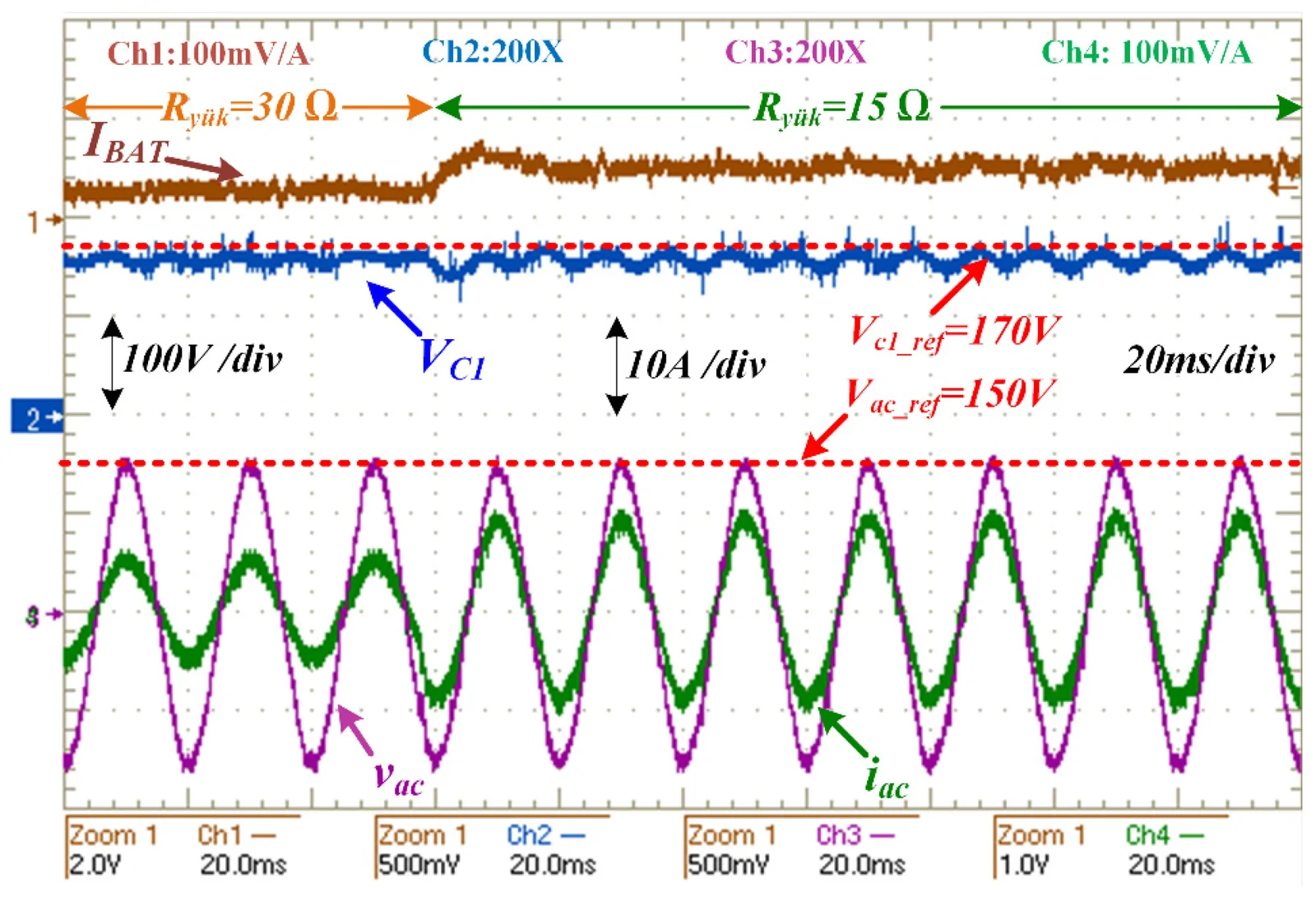
Dynamic responses obtained under a step change in load with the proposed hybrid control method for *K_p_* = 0.4 and *K_i_* = 30.

**Figure 8:**
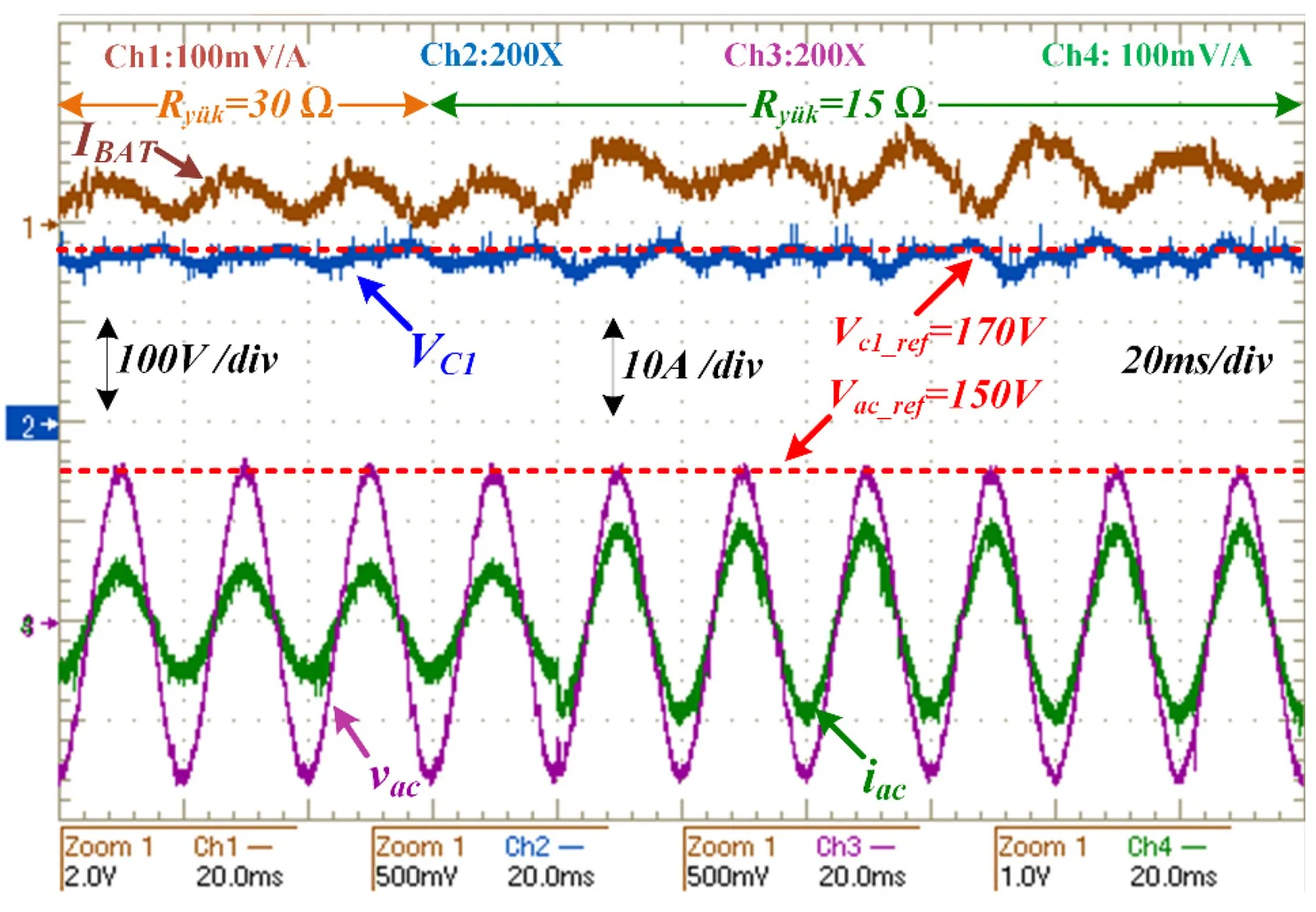
Dynamic responses obtained under a step change in load with the proposed hybrid control method for *K_p_* = 0.7 and *K_i_* = 30.

In the proposed hybrid control method, a control design application is implemented using PI control for the DC side and PR control for the AC side. Both controller-parameter selections were done using a two-step procedure. To identify the preliminary parameter-setting range, small-signal modeling and Bode plot techniques were used to ensure gain at the resonant point and proper stability margins. Each of these preliminarily determined sets of parameters was then evaluated based on three performance criteria: steady-state error of the controlled variable, THD (total harmonic distortion) of the output voltage less than 4%, and settling time under load-step perturbations. The final set of parameter values shown in Table 1 best satisfies all three criteria. The PI control method controls the capacitor voltage *C*_1_ in the impedance circuit, while the PR control method controls the output voltage on the AC side. The effectiveness of the PI control method is demonstrated by the results obtained at different control parameters, shown in Figure 7 and Figure 8. Accordingly, Figure 7 shows the dynamic responses of the *V_C_*_1_ and *I_BAT_* variables on the DC side, and the *v_ac_* and *i_ac_* variables on the AC side, as the load resistance is decreased from 30ω to 15ω, with the *K_p_* parameter set to 0.4 in the PI control method that controls the capacitor voltage *C*_1_ on the DC side of the topology. While the input voltage is 140 V, the PI controller sets the *V_C_*_1_ capacitor voltage to 170 V. The inverter output voltage is set to 150 V by the PR control method. The system was tested with different control parameters to evaluate its performance. When the load connected to the inverter output decreases from 30ω to 15ω, jumps in *I_BAT_* current at the 2ω frequency, a collapse in *V_C_*_1_ voltage, steady-state error, and distortion in *I_BAT_* current are clearly seen in Figure 7. The low-frequency ripples in the *V_C_*_1_ voltage are transferred to the I_BAT_* reference current parameter, as shown in the block diagram. Since the *I_BAT_* current follows the I_BAT_* reference current, the magnitude of the low-frequency ripples in the *I_BAT_* current signal decreases in direct proportion to the decrease in the magnitude of the low-frequency ripples of the I_BAT_* reference current signal. Figure 7 shows that when *K_p_* = 0.4, the current through the load is approximately doubled by reducing the load resistance from 30 Ω to 15 Ω. In addition to the increase in *i_ac_* current with changes in load resistance, Figure 7 clearly shows that the inverter output voltage maintains system stability at the reference voltage level (150 V) thanks to the PR controller in the topology. Since the effect of the current value drawn by the load from the input source is directly reflected on the *L*_1_ coil current, the control parameters *K_p_* and *K_i_* of the PI control method that provides the control of the *C*_1_ capacitor voltage should be determined at the optimal level. In the result graph shown in Figure 7, with PI control parameters *K_p_* = 0.4 and *K_i_* = 30, when the load connected to the inverter output is reduced from 30ω to 15ω, the *V_C_*_1_ voltage is 170 V, while it is controlled at 150 V during the load change. However, a steady-state deviation occurs in the *V_C_*_1_ voltage at approximately 15 V due to collapse and oscillation. The system reaches steady state in approximately 20 ms. The *I_BAT_* coil current is approximately doubled. The ripples component in *V_C_*_1_ at 2ω almost doubles after the load change. It can be seen that the proposed hybrid control structure provides DC-side control with steady-state error. Although there are abrupt changes in *i_ac_* current under linear load, the output voltage is sinusoidal with an amplitude of 150 V. As shown in Figure 7, the *v_ac_* voltage corresponds to the inverter’s maximum output voltage. The results show that the proposed hybrid control structure successfully achieves AC-side control, ensuring that the controlled variables track their references accurately.

The DC-side response is more sensitive to the load disturbance. The capacitor voltage (*V_C_*_1_), whose reference is 170 V, exhibits an approximately 15 V deviation following the load transition. This corresponds to a normalized voltage deviation of approximately 8.8% with respect to the reference value. The response reaches its new steady operating condition in approximately 20 ms. However, because (*V_C_*_1_) does not completely recover to its reference value, the results indicate insufficient DC-side regulation for (*K_p_* = 0.4) and (*K_i_* = 30) under this severe load disturbance.

The battery-current response also increases approximately twofold following the load transition and exhibits increased low-frequency ripple. This behavior indicates that the abrupt increase in AC-side power demand is transferred to the battery-assisted impedance network. Therefore, although the selected gains provide a relatively fast transient response, the observed capacitor-voltage deviation and battery-current ripple demonstrate that (*K_p_* = 0.4) does not provide the best compromise between transient speed and DC-side regulation accuracy. These results motivate evaluating a higher proportional gain and conducting a systematic gain sensitivity analysis.

Figure 8 illustrates the dynamic responses of the DC- and AC-side variables when the load resistance decreases from 30 Ω to 15 Ω under PI control, with *K_p_* = 0.7 and *K_i_* = 30. The voltage *V_C_*_1_ follows the reference voltage value of 150 V. However, low-frequency ripples and distortions occur in the *I_BAT_* current. This means that 1 A of current is unnecessarily drawn when a 30ω load is connected to the inverter output, and approximately 2 A is drawn when a 15ω load is connected, due to low-frequency ripple and distortion.

As the current drawn from the source increases, the amount of current drawn unnecessarily increases. Additionally, these ripples also reduce system efficiency. Figure 8 illustrates that the distortion on the DC side does not affect the AC side. Although the load at the inverter output is reduced and the current drawn is approximately doubled, the transition is linear, and the output voltage is sinusoidal with an amplitude of 150 V. As shown in Figure 8, the *v_ac_* voltage corresponds to the inverter’s maximum output voltage.

**Figure 9:**
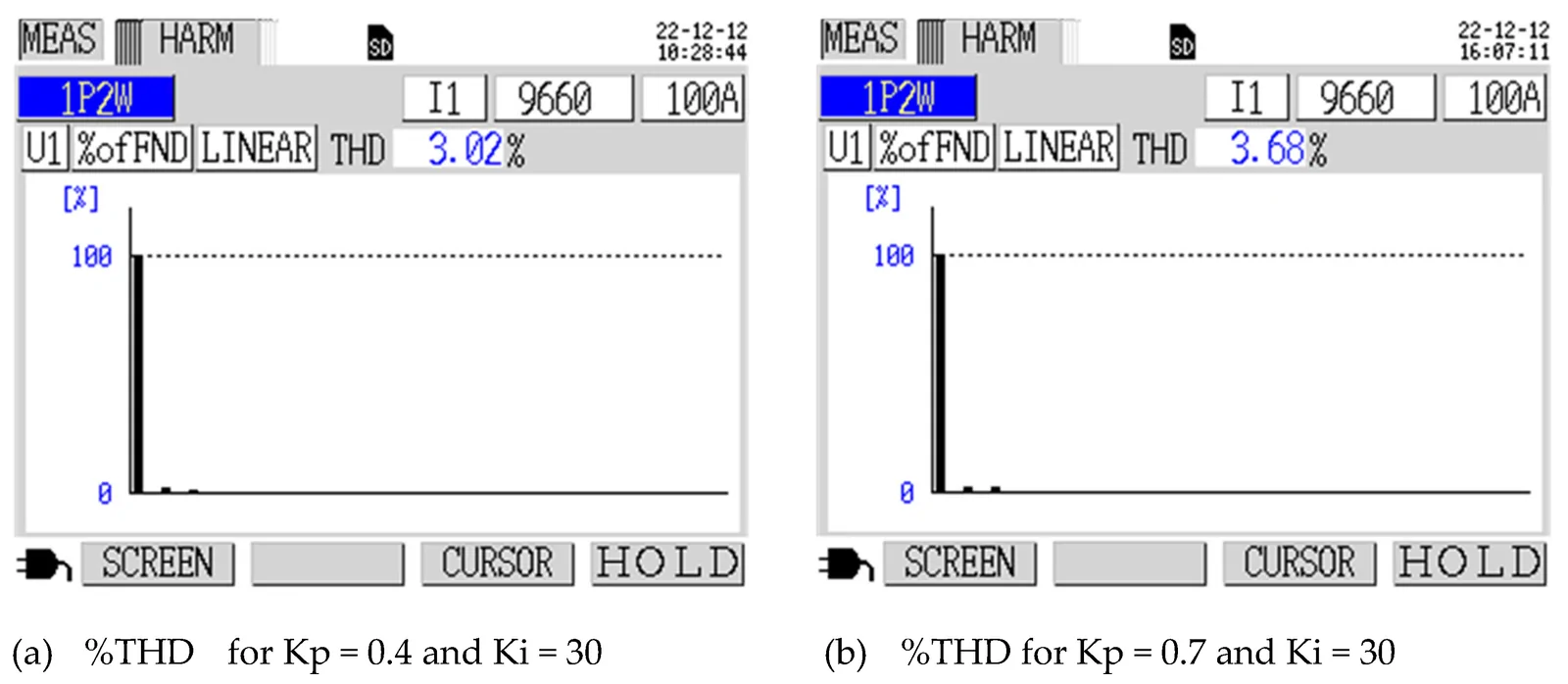
%THD with the proposed hybrid control method for *K_p_* = 0.4 and *K_i_* = 30 and *K_p_* = 0.7 and *K_i_* = 30.

In addition to the inverter output-voltage regulation, the magnitude of the harmonic components is also an important factor. As shown in Figure 9a, the harmonic distortion of the output voltage is 3.02% with *K_p_* = 0.4 and *K_i_* = 30, and in Figure 9b, it is 3.68% with *K_p_* = 0.7 and *K_i_* = 30. From the load current (*i_ac_*) results, it is clear that the linear load draws a sinusoidal current from the inverter, and the load voltage has a sinusoidal waveform and follows its reference. Figure 7 and Figure 8 show the steady-state results for different *K_p_* and *K_i_* parameters. According to the THD results in Figure 9a,b for these parameters, the optimal selection of PI control parameters directly affects the system results. Since the steady-state error in the system is undesirable and negatively affects all system parameters, the *K_p_* parameter is set to 0.7 and the Ki parameter to 30. It aims to eliminate the low-frequency ripples, which are a negative side effect of this selection.

**Figure 10:**
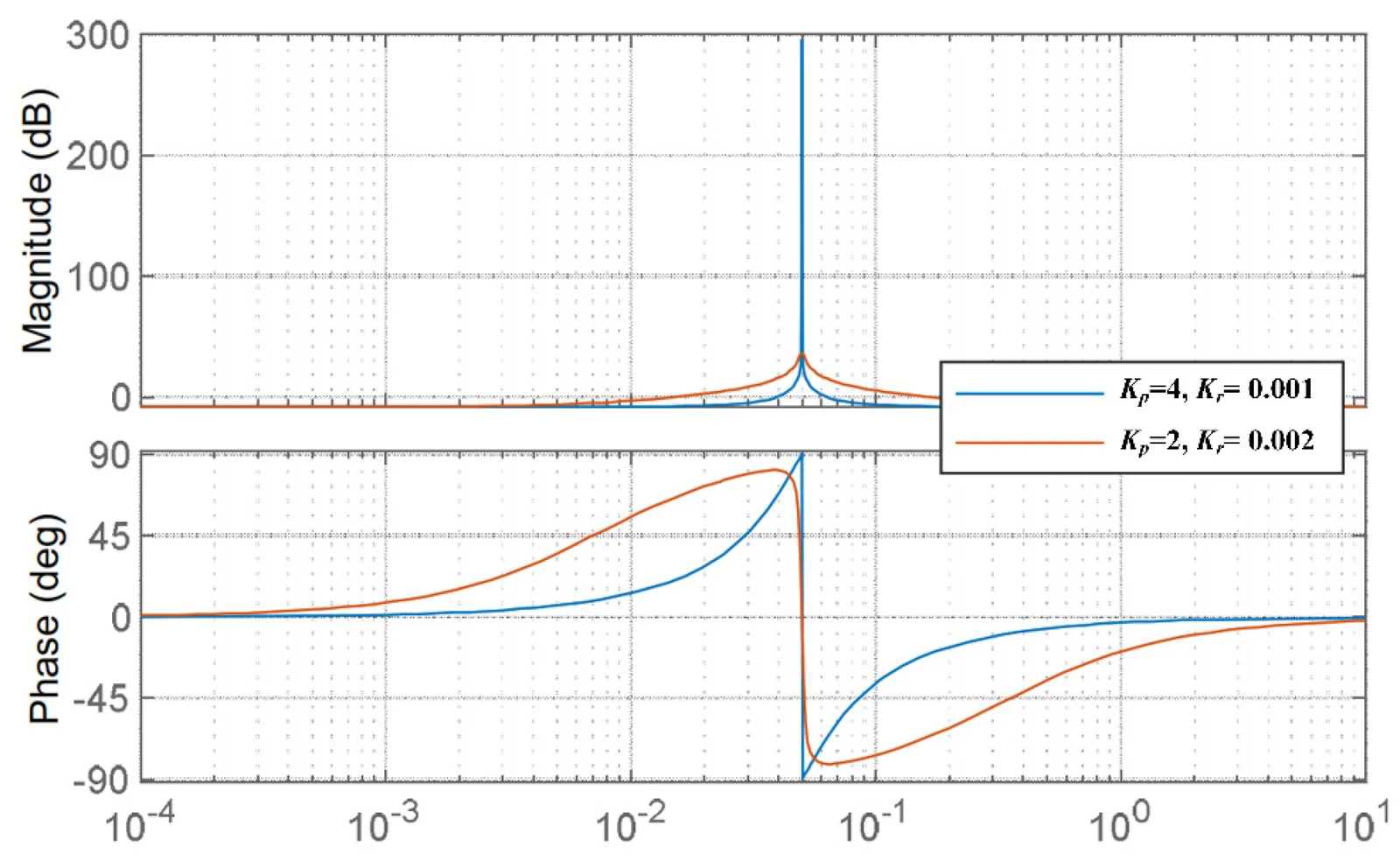
The Bode plot of the PR control method for different *K_p_* and *K_r_* parameter values.

The equation of the PR controller is given below,

(14) $$G_{PR}\left(s\right)=K_{p}+\frac{2K_{r}\omega_{c}s}{s^{2}+2\omega_{c}s+\omega^{2}}$$

where ***ω****ω*_0_ defines the fundamental angular frequency, and *K_p_* and *K_r_* parameters are proportional and resonant coefficients. The *K_p_* parameter is selected like the PI control method to achieve the desired phase and gain margins. The ideal resonant controller has infinite gain at the resonant frequency *ω*_0_. This feature prevents the controller implementation in reality. The PR control method behaves as a high-gain, low-pass filter with finite gain and a wider bandwidth. The Bode plot of the PR control method for *K_p_*:2, *K_r_*:0.002, and *K_p_*:4, *K_r_*:0.001 is shown in Figure 10. The *K_p_* parameter is defined as 2, and the Kr parameter is adjusted as 0.002 in the PR controller to ensure that the inverter output voltage is a pure sine wave.

The comparative results obtained for different PI-controller gains are summarized in Table 2. The integral gain was maintained at (*K_i_* = 30), while the proportional gain was varied between (*K_p_* = 0.4) and (*K_p_* = 0.7) to evaluate its influence on the DC-side dynamic performance. For (*K_p_* = 0.4), the load transition from 30 Ω to 15 Ω results in an approximately 15 V deviation in the capacitor voltage (*V_C_*_1_), corresponding to approximately 8.8% of the 170 V reference value. Although the response reaches its new operating condition in approximately 20 ms, a noticeable steady-state deviation and increased low-frequency ripple in the battery current are observed.

Increasing the proportional gain to (*K_p_* = 0.7), while maintaining (*K_i_* = 30), improves the capacitor-voltage tracking performance and reduces the steady-state regulation error. However, low-frequency ripple and distortion remain observable in the battery-current response. On the AC side, both controller settings maintain a sinusoidal output voltage at approximately its reference value, indicating that the PR controller successfully preserves AC-side voltage regulation during the load disturbance.

The harmonic results introduce an additional trade-off in controller selection. The output voltage THD is 3.02% for (*K_p_* = 0.4) and 3.68% for (*K_p_* = 0.7). Thus, although the lower proportional gain provides slightly better harmonic performance, it results in inferior DC-side voltage regulation. Consequently, (*K_p_* = 0.7) and (*K_i_* = 30) are selected as the nominal PI-controller parameters because they provide a more appropriate compromise between DC-side voltage tracking accuracy, transient performance, and acceptable AC-side harmonic distortion.

## 5. Experimental Results

**Figure 11:**
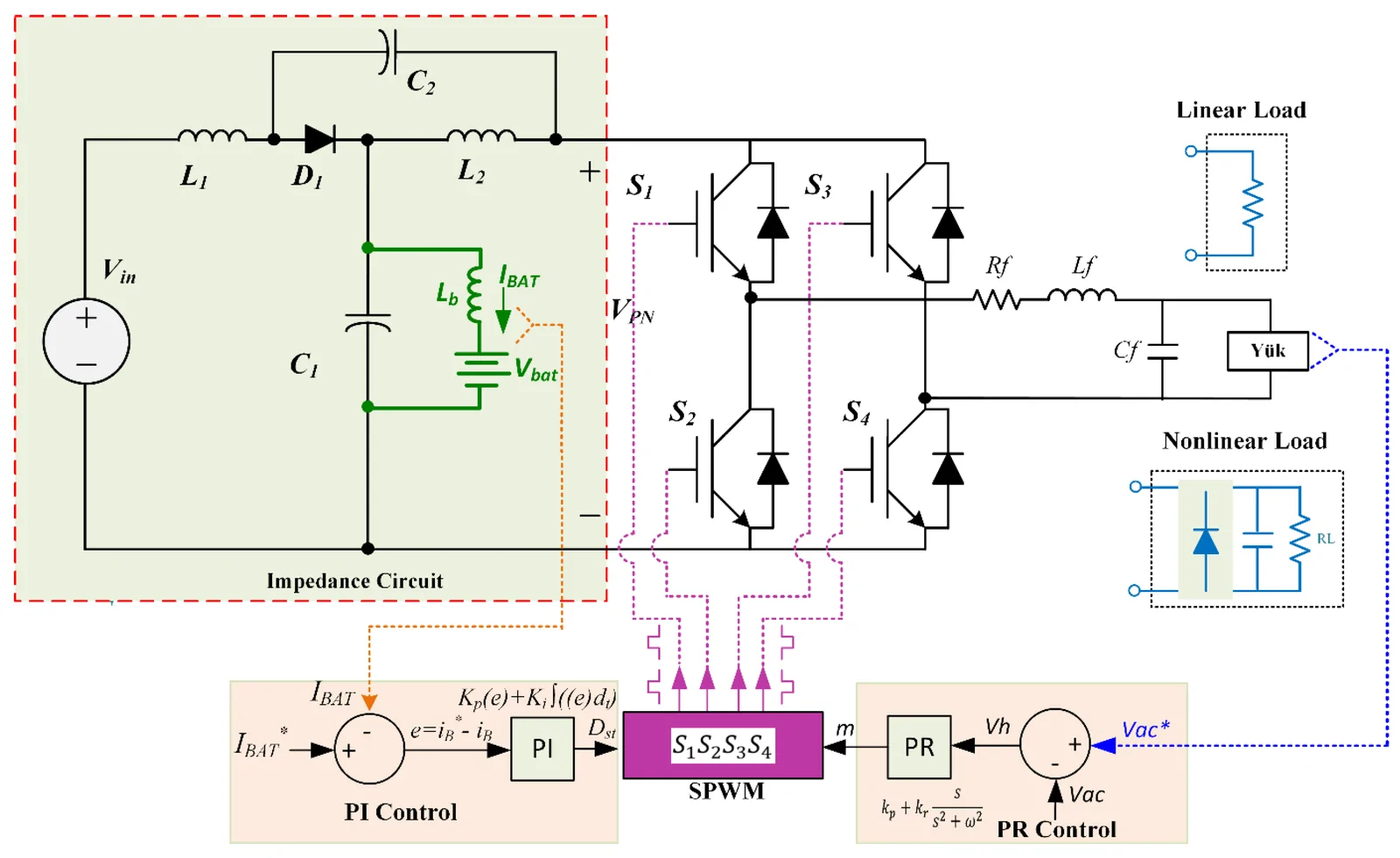
Block scheme of the proposed hybrid control method.

This section presents experimental studies and results to validate the proposed control method. Also, steady-state and dynamic response results are presented to demonstrate the performance of standalone mode operation. Thus, the performance and applicability of the control method are established. The block scheme of the proposed control method is given in Figure 11.

**Figure 12:**
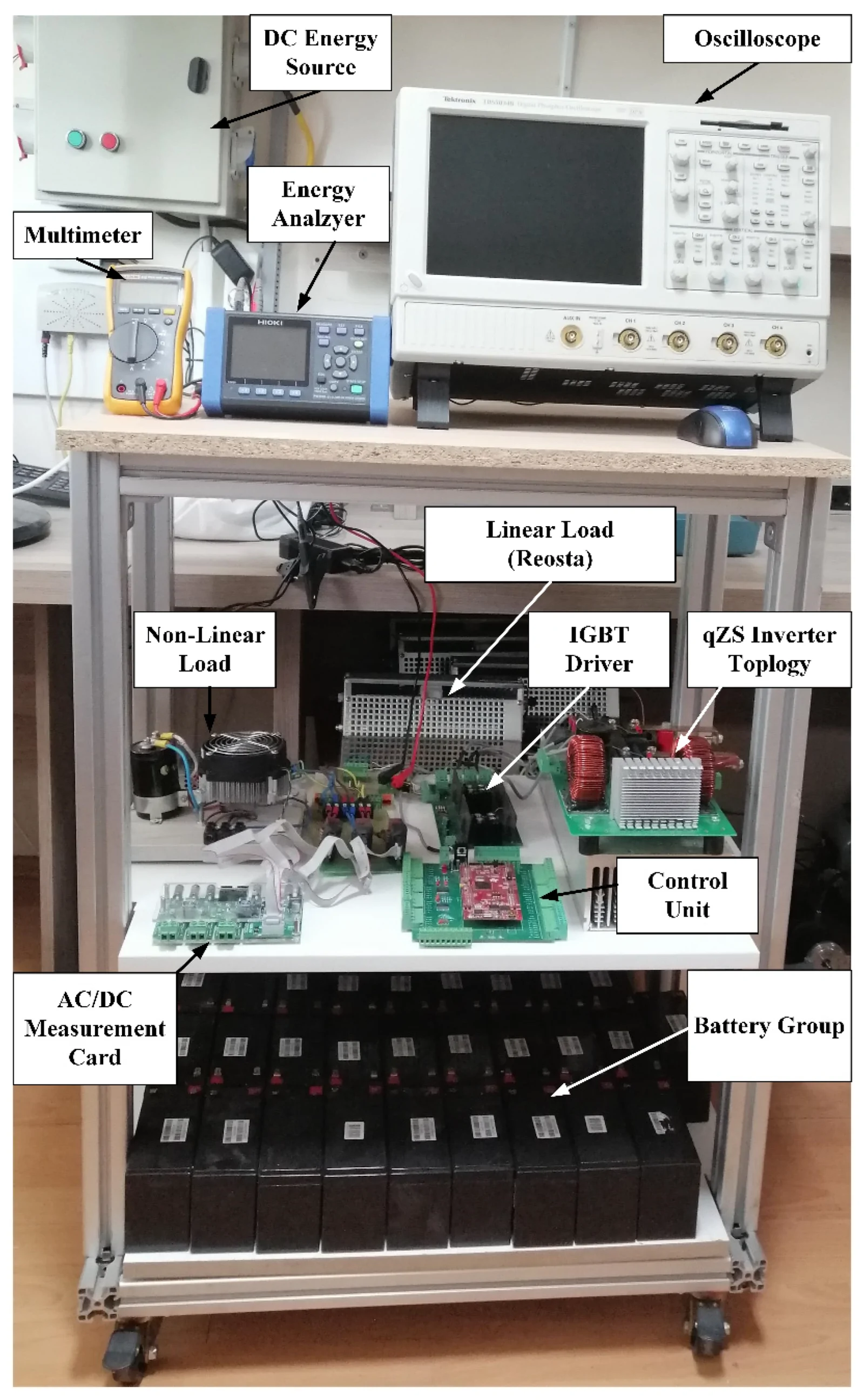
Experimental setup.

The laboratory prototype of the battery-assisted qZSI is shown in Figure 12 to validate the proposed design procedure. The components of the experimental setup include an IGBT driver circuit, an impedance circuit, current and voltage measurement units, an LC filter, a main board, a load, and an inverter circuit. A Texas 28F379D Launchpad-based control board, purchased from Texas Instruments in the USA is used as the main control unit in experimental results. The application prototype of the battery-assisted qZSI is designed with *L*_1_ = *L*_2_ = 1.5 mH and C_1_ = C_2_ = 1000 µF, using an IGBT. Fuji-2MBI150L-120 series IGBTs, purchased from Fuji Electric in the Japan are used in the inverter layer of the single-phase battery-assisted qZSI topology. The VLA-502-01 drive circuit drives the IGBTs. In the proposed topology, the Taraz-USM3IV series isolated voltage and current-sensing module, purchased from Taraz Technologies in the Pakistan is used to measure voltage and current. A 30 Ω ohmic load is connected to the inverter’s output. A Magna Power 6 kW DC source, purchased from Magna-Power Electronics in the USA is used as the input voltage for the battery-assisted qZSI. In addition, an LC filter consisting of a 2 mH inductor and a 20 µF capacitor is connected to the inverter’s output.

The experimental setup was designed for 1 kW. Twenty-eight batteries with 12 V, 9 A capacity were used. Fourteen batteries were connected in series to form two separate groups, then connected in parallel to form a 170 V battery bank. This battery bank is connected in parallel to capacitor *C*_1_. Lithium-ion battery types were used in the work. The nominal battery charging current is approximately 5 A.

### 5.1. Steady-State Analysis Results

**Figure 13:**
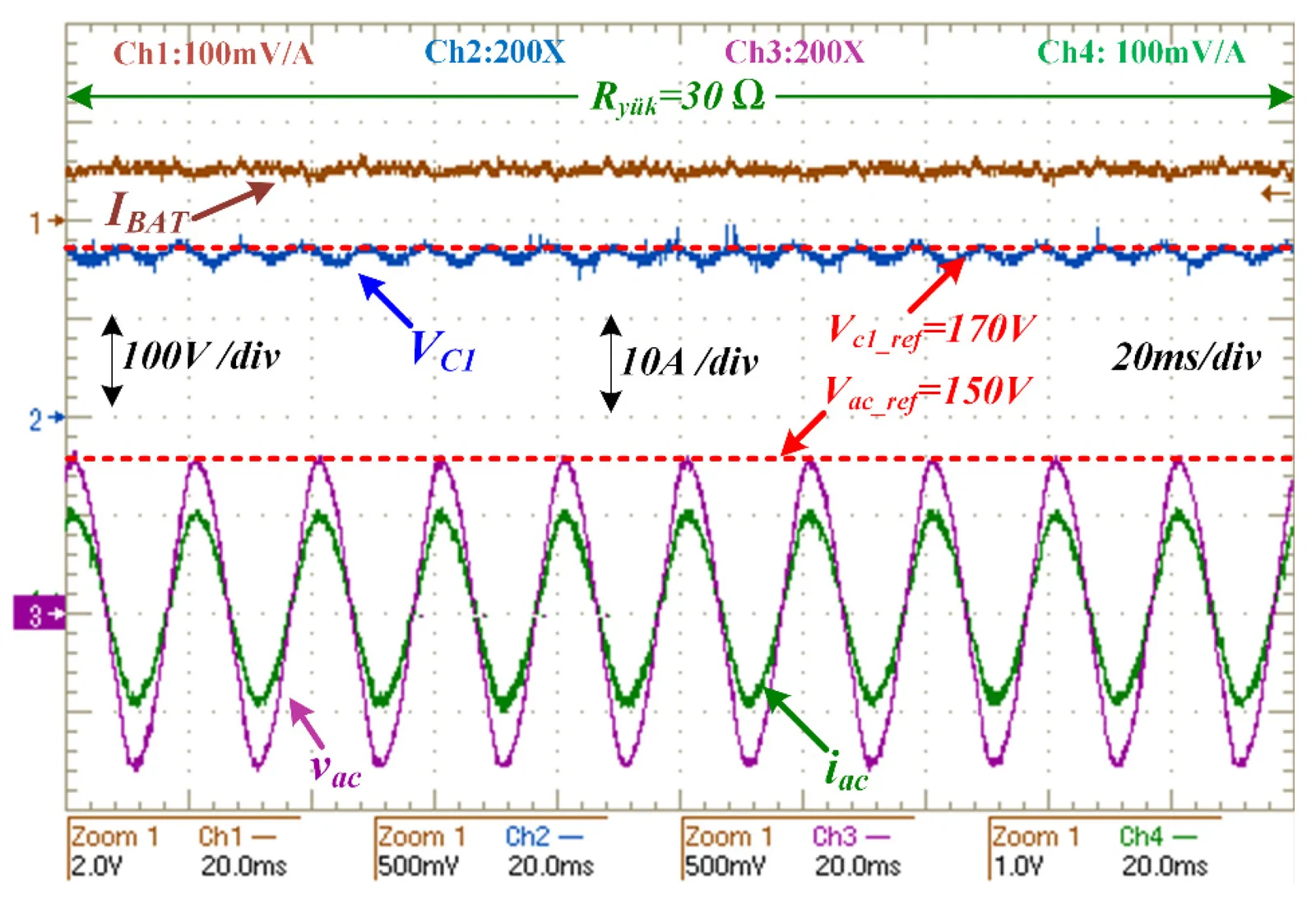
Application results for steady-state analysis of the battery-assisted qZSI.

Figure 13 shows the steady-state analysis results for the proposed control method in standalone mode operation of the battery-assisted qZSI. In this study, the input voltage is set to 140 V; the load resistance is 30 Ω; the reference value of capacitor voltage C_1_ (V_C1_*) is set to 170 V; and, finally, the output voltage (*v_ac_*) is set to 150 V. As shown in the figure, *V_C_*_1_ is successfully set to its reference value by the DC-side control method. Furthermore, the capacitor voltage *V_C_*_2_ is measured at 30 V and the *V_PN_* voltage at 200 V, which is approximately the sum of the voltages *V_C_*_1_ and *V_C_*_2_. The boost factor and short-circuit mode duty ratio of the topology are approximately 1.875 and 0.233, respectively. The inverter’s output voltage is stabilized at 150 V with the PR controller method. *V_ac_* represents the inverter’s maximum output voltage. Thus, the proposed control methods ensure stable operation of the battery-assisted qZSI for the reference values. To show the steady-state response of the proposed system, the input voltage is set to 140 V, and the battery charging current (*I_BAT_*) is set to 5 A. Figure 13 shows the steady-state waveforms of the *C*_1_ capacitor voltage, battery current, inverter output voltage, and current.

**Figure 14:**
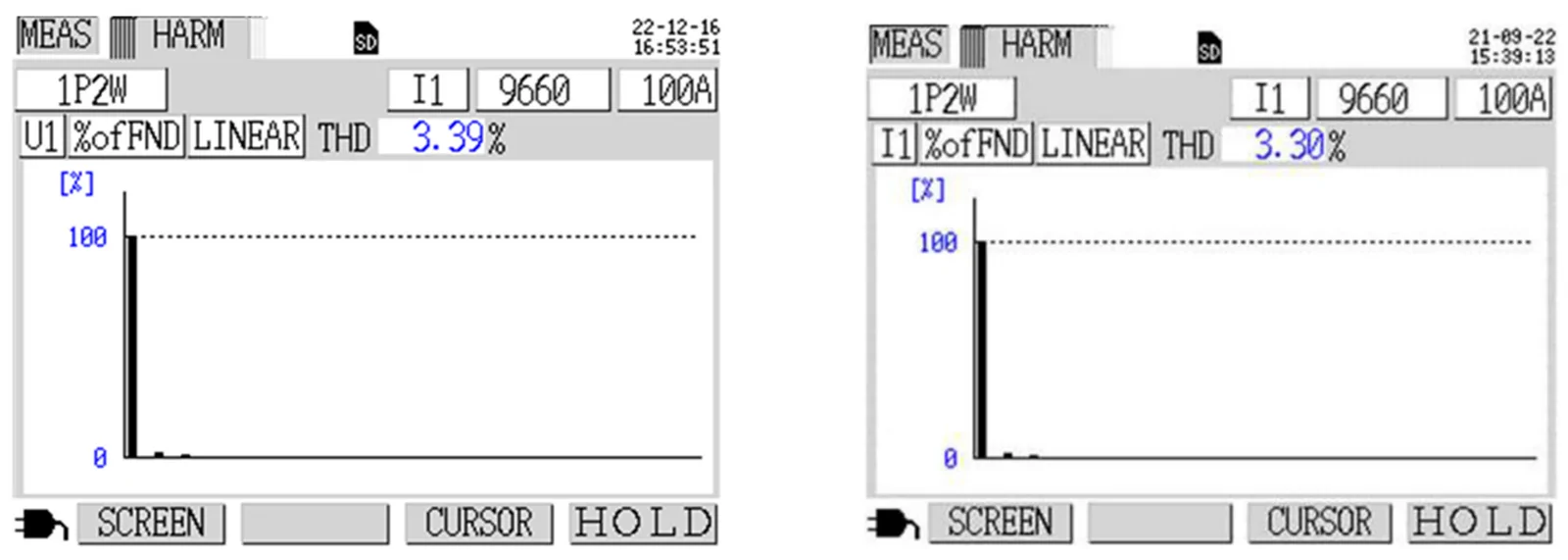
Voltage and current harmonic results under linear load.

Voltage and current harmonic results under linear load are shown in Figure 14. The total harmonic voltage distortion of the output voltage is 3.39%. As shown in the load current results, the linear load draws a non-sinusoidal current from the inverter. The harmonic distortion in the load current is 3.30%. Under these conditions, it is clearly seen that the load voltage has a sinusoidal waveform and is regulated to its reference.

### 5.2. Dynamic State Analysis Results

**Figure 15:**
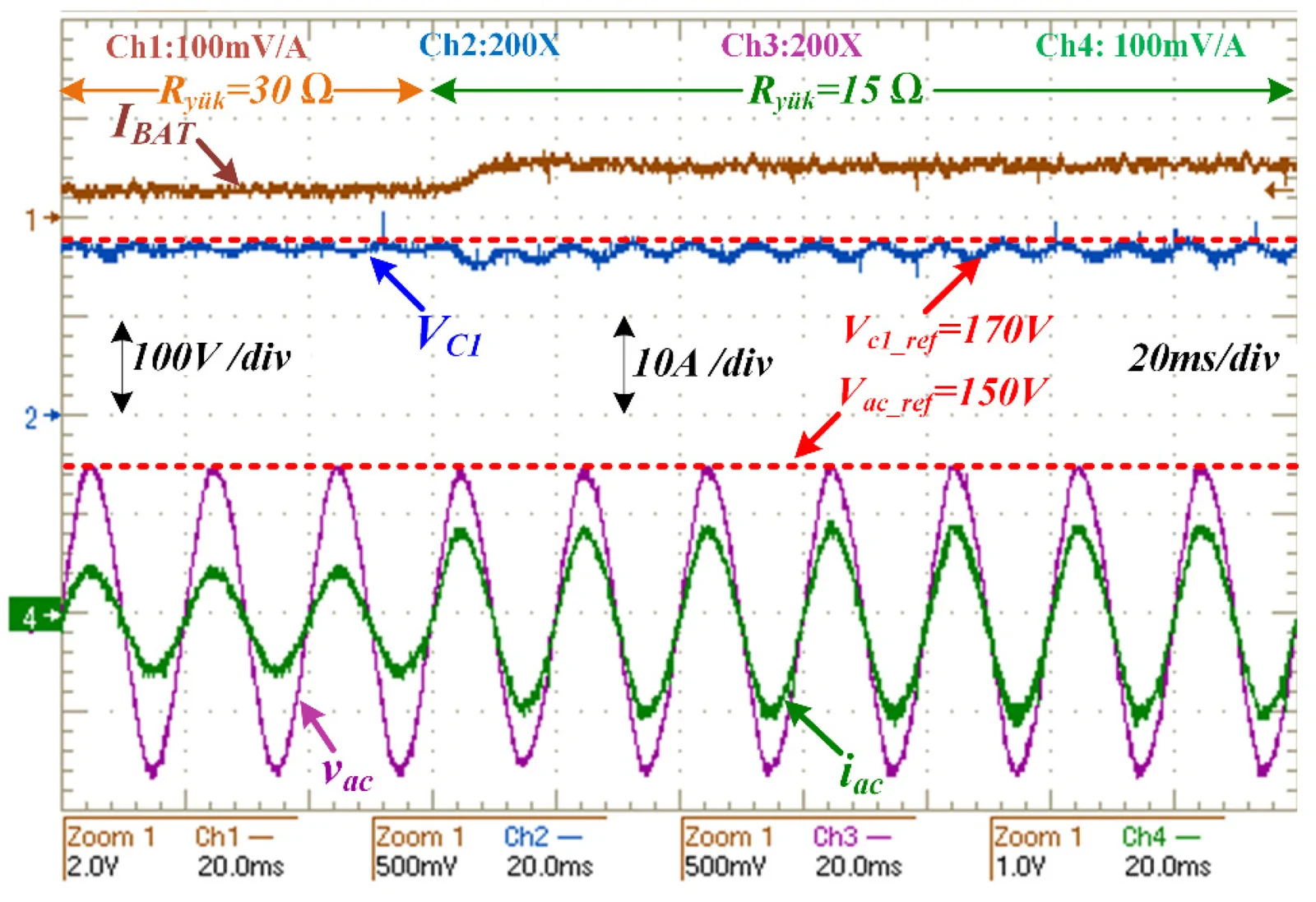
Dynamic responses obtained under a step change in load with the proposed hybrid control method when the load is reduced from 30 Ω to 15 Ω.

Three different scenarios are presented for the dynamic response results to verify the effectiveness of the DC- and AC-side controllers. In the first scenario, the input voltage (*V_in_*) and the capacitor *C*_1_ reference voltage (*V_C_*_1_*) were kept constant at 140 V and 170 V, respectively, while the load was reduced from 30 Ω to 15 Ω. The results obtained under this transition are shown in Figure 15. When the voltage step change occurred, the load current on the AC side increased from 2.5 A to 5 A. Despite disturbances in the DC- and AC-side currents, it is clear that a steady-state deviation is occurring, with the *V_C_*_1_ voltage oscillating at approximately ±15 V. The V_C1_ voltage cannot track the reference voltage of 170 V.

Figure 15 presents the experimental response of the proposed controller to a step change in load resistance from 30 Ω to 15 Ω. The abrupt reduction in load resistance substantially increases the power demanded from the AC side and causes a transient redistribution of energy among the battery and the passive elements of the qZSI impedance network. Although the AC output voltage remains regulated during the disturbance, a residual low-frequency oscillation is observed in the DC-side capacitor voltage (*V_C_*_1_).

In particular, (*V_C_*_1_) exhibits an approximately ±15 V variation around its 170 V reference, corresponding to a normalized deviation of approximately ±8.8%. Therefore, the response should not be interpreted as zero-error capacitor-voltage regulation. Rather, the result indicates that the proposed PI controller maintains bounded DC-side operation but has limited disturbance-rejection capability for the capacitor-voltage dynamics under this severe load transition. The battery current also changes in response to the increased load demand, reflecting the redistribution of power within the battery-assisted impedance network.

In contrast, the AC-side output voltage remains close to its reference and retains its sinusoidal waveform, demonstrating that the PR-controlled AC voltage loop is less affected by the investigated load disturbance. Hence, the experimental results reveal different disturbance rejection characteristics of the DC- and AC-side control loops: satisfactory AC-side voltage regulation is maintained, whereas a finite residual deviation remains in the DC-side capacitor voltage.

**Figure 16:**
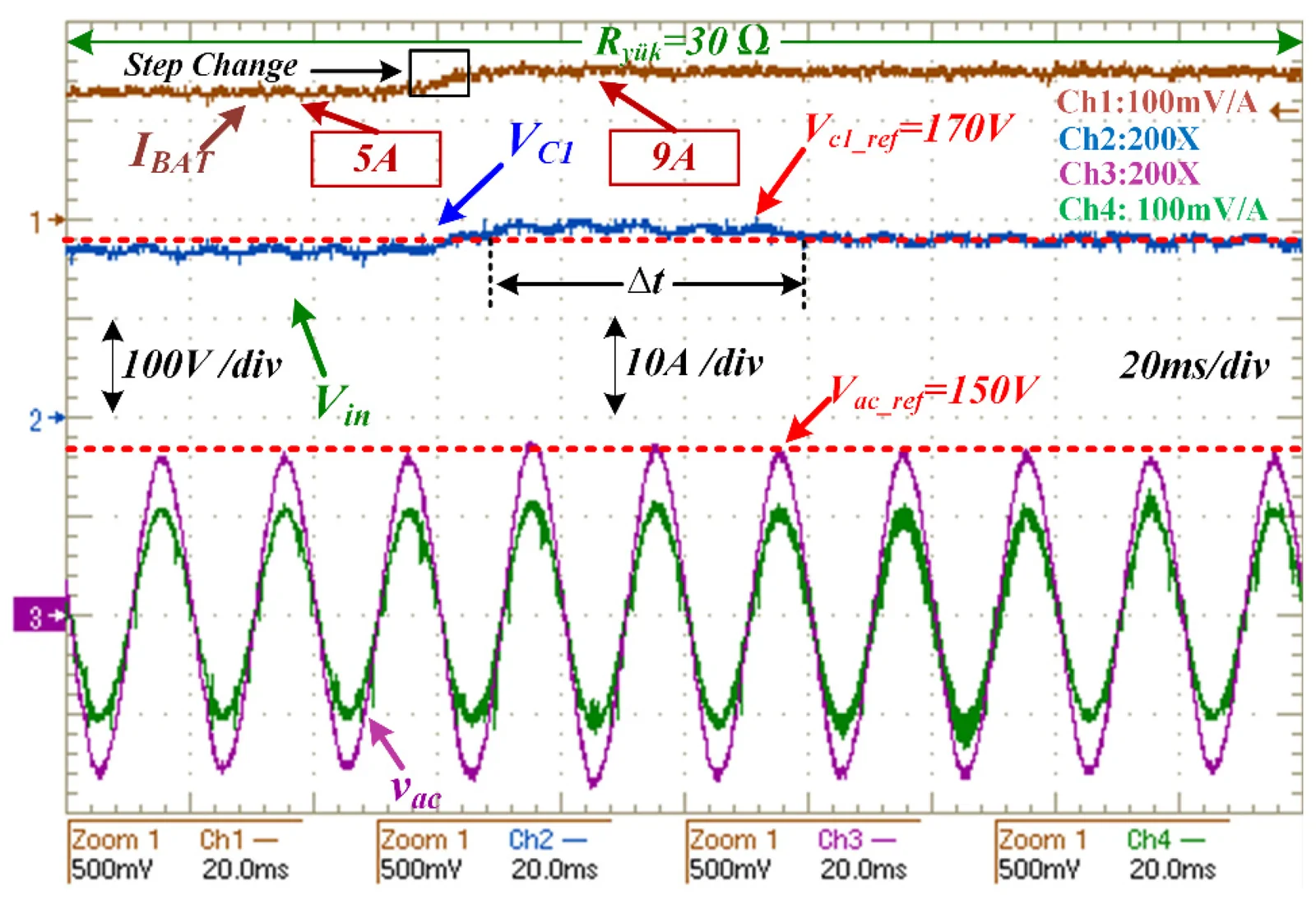
Dynamic responses obtained under a step change in battery current with the proposed hybrid control method when battery current is increased from 5 A to 9 A.

In the second operating state, the capacitor reference voltage *C*_1_ (*V_C_*_1_*) and the output voltage *V_ac_* are kept constant at 170 V and 150 V, respectively, while the battery current increases from 5 A to 9 A. In this case, the *v_ac_* output voltage operates at a constant value of the reference output voltage (*v_ac_ref_*) for the AC side of the qZSI. When the battery current increased to 9 A, the capacitor voltage V_C1_ increased from 170 V to 180 V during the 50 ms transition period. However, the *V_C_*_1_ voltage is kept constant at the reference values, except during the 50 ms transition period due to the input voltage change. The implementation results for the second operating state are shown in Figure 16. Here, “*v_ac_ref_*” represents the reference output-voltage value for the AC side of the battery-assisted qZSI, while v_ac_ represents the maximum output voltage of the inverter.

**Figure 17:**
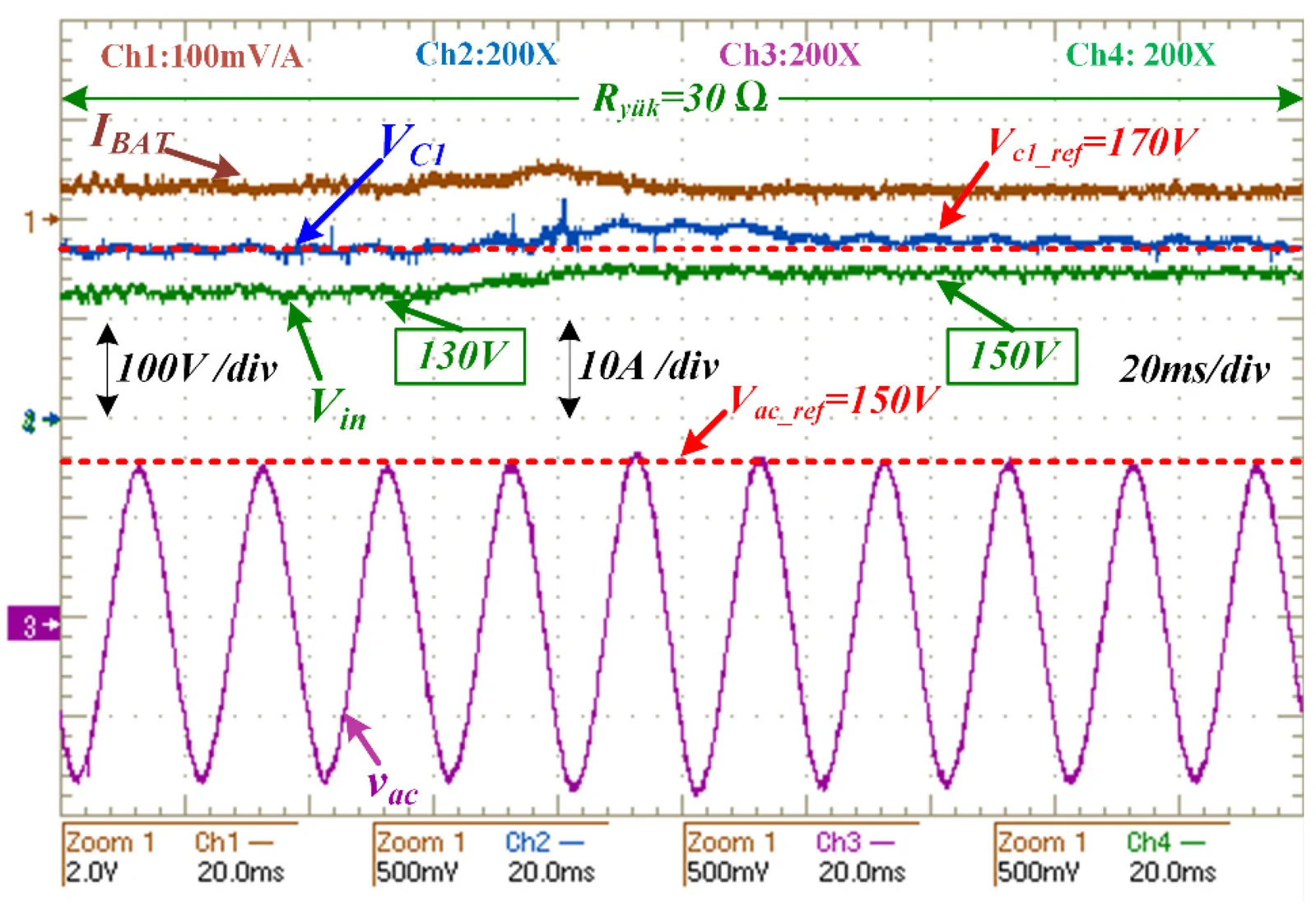
Dynamic responses obtained under a step change in battery current with the proposed hybrid control method when V_in_ voltage is increased from 130 V to 150 V under a linear load.

In addition to the second operating state, the dynamic response of the *I_BAT_* current when the input voltage is changed from 130 V to 150 V is shown in Figure 17. The *V_C_*_1_ capacitor voltage and the vac output voltage are kept constant at the reference voltage, with +/−15 V oscillation, by PI and PR controllers. *I_BAT_* current is approximately 3 A. *V_C_*_1_ stabilizes at about 50 ms (∆t), and the v_ac_ output voltage is kept constant at the reference voltage value by the PR controller. As shown in Figure 17, the *v_ac_* voltage corresponds to the inverter’s maximum output voltage.

**Figure 18:**
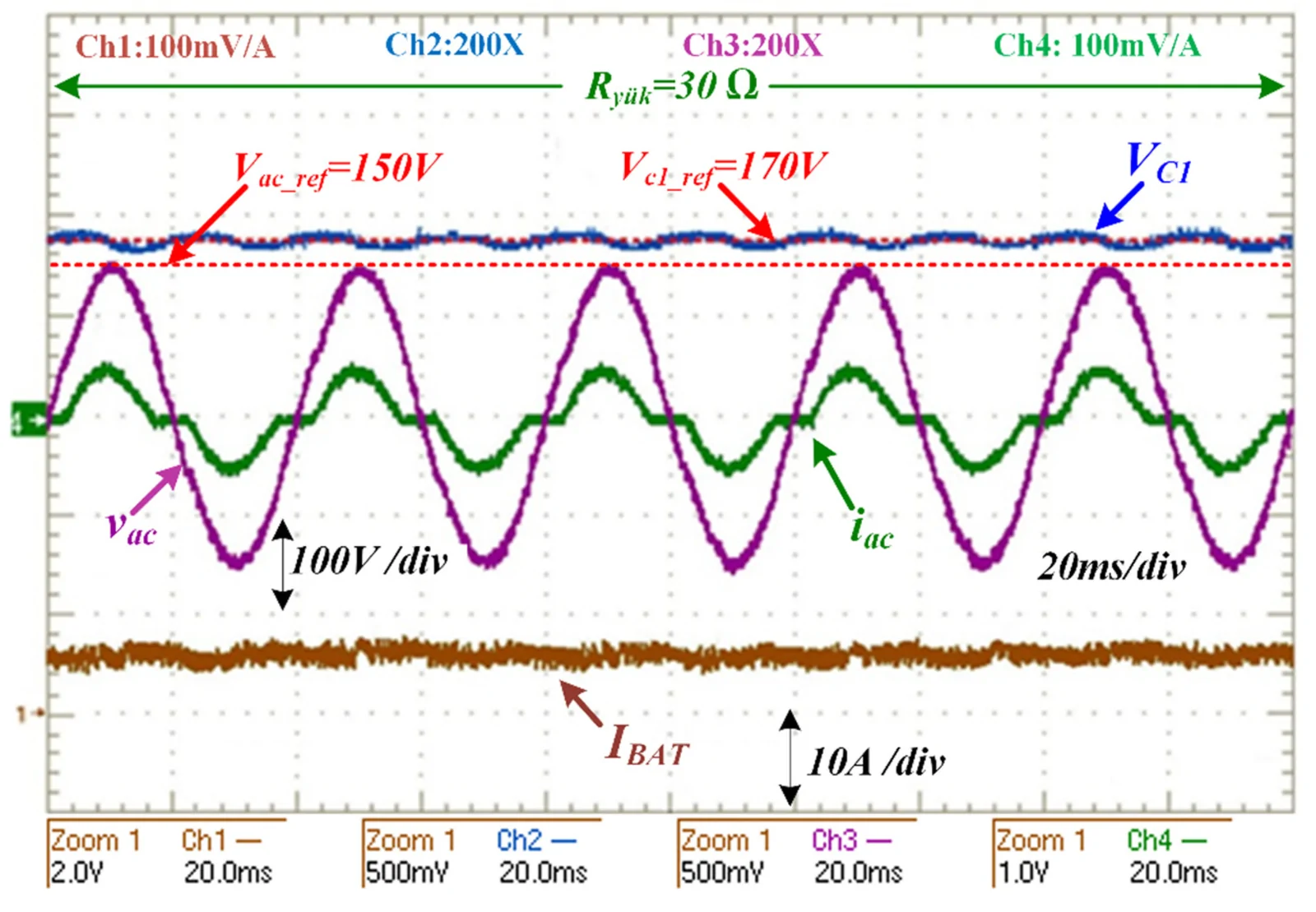
Steady-state results with the proposed hybrid control method under nonlinear load.

**Figure 19:**
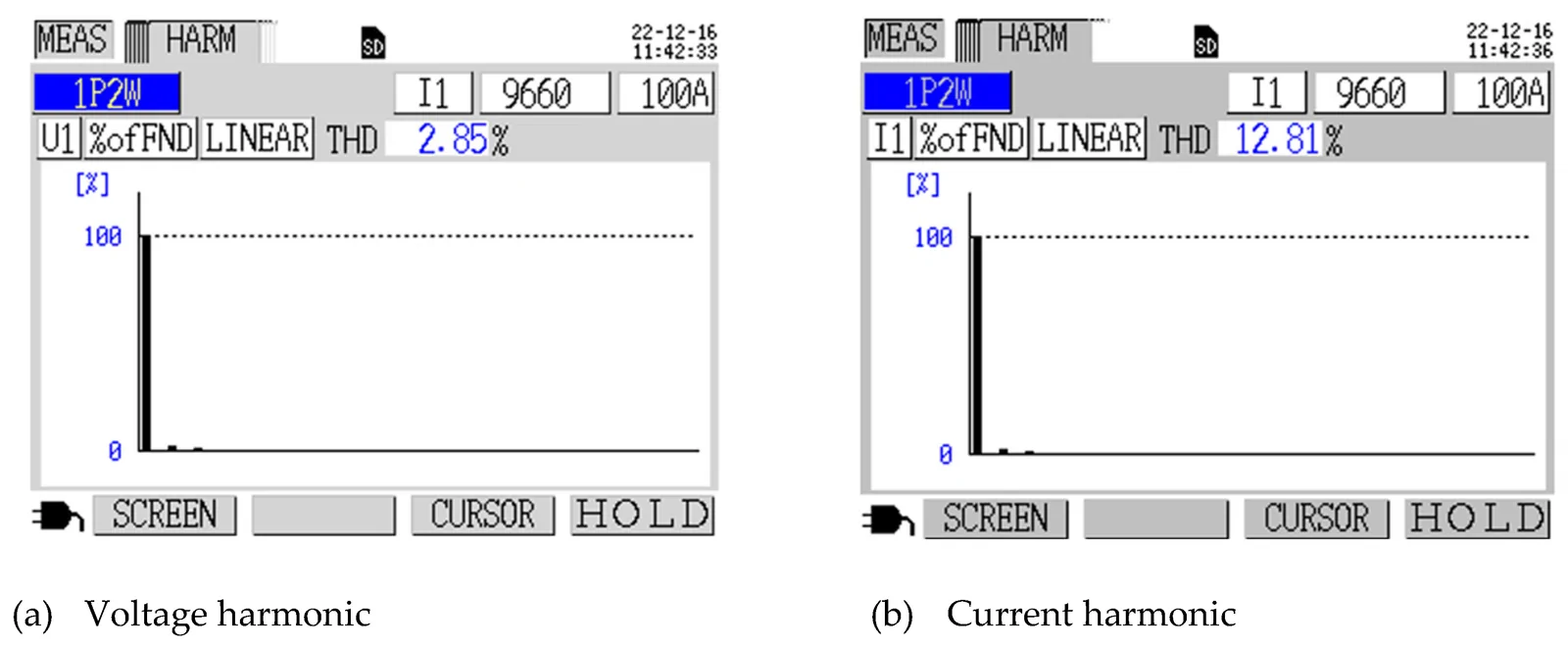
Voltage and current harmonic results under a nonlinear load.

Figure 18 shows the steady-state results of the inverter output voltage (*v_ac_*), capacitor voltage (*V_C_*_1_), load current (*i_ac_*), and battery current (*I_BAT_*) under a nonlinear load, and Figure 19 shows the harmonic results in this case. As shown in the load current (*i_ac_*) result, the nonlinear load draws a non-sinusoidal current from the inverter. As shown in Figure 19b, the load current harmonic distortion is 12.81%. In this case, it is clearly seen that the load voltage has a sinusoidal waveform and is regulated to its reference. Furthermore, the result in Figure 19a shows that the total harmonic content of the load voltage is as low as 2.85%. From the steady-state results in Figure 13 and Figure 18, it can be concluded that voltage regulation is achieved with low harmonics for both linear and nonlinear loads.

Table 3 compares the main control and implementation characteristics of the proposed battery-assisted qZSI with the related methods reported in [28,29,30,31]. To ensure a transparent and fair comparison, the revised table distinguishes qualitative system characteristics from quantitative performance indices. A parameter that is not explicitly reported in a reference is denoted as “NR (Not Reported)” rather than being interpreted as inferior performance. In particular, voltage and current THD values are not explicitly reported in [28,29,30,31]; therefore, these studies cannot be quantitatively ranked against the proposed method in terms of harmonic performance. The experimentally measured voltage and current THD values of the proposed system, 3.39% and 3.30%, respectively, are therefore reported only as experimentally verified characteristics and are not used to claim superiority over studies for which equivalent data are unavailable. For dynamic-performance assessment, common quantitative indices are defined as settling time, percentage overshoot, and steady-state error. These indices are included only when they are explicitly reported under sufficiently comparable operating conditions. When these values are unavailable in a reference, the corresponding entries are indicated as NR. For the proposed controller, the indices are evaluated from the experimental transient responses using identical mathematical definitions. It should also be noted that the compared studies do not address identical operating objectives. References [28,29] primarily consider grid-connected photovoltaic systems with battery energy storage, whereas the present work focuses on standalone operation. Reference [30] focuses on power-based battery-current control, while [31] develops a state-space feedback battery-current controller. Consequently, the revised comparison avoids unsupported direct performance rankings and instead highlights the experimentally demonstrated characteristics, disturbance scenarios, implementation complexity, and quantitative performance of each method where comparable information is available.

The quantitative dynamic performance indices obtained from the experimental responses are summarized in Table 4. Unlike a qualitative evaluation based solely on transient waveforms, the use of settling time, overshoot, and steady-state error provides a consistent basis for assessing the transient behavior of the proposed hybrid controller under various operating disturbances. Under the investigated load, battery current, and input-voltage disturbances, the capacitor voltage and battery-current responses exhibit different transient characteristics because the disturbances affect the energy balance of the impedance network in different ways. In particular, the capacitor-voltage response reflects the ability of the DC-side controller to restore the energy balance of the qZSI impedance network, whereas the battery-current response indicates the effectiveness of battery-current regulation during transient operation. The results in Table 3 show that the selected PI+PR controller parameters provide an acceptable compromise between transient speed, overshoot, and steady-state tracking accuracy for the investigated operating conditions. The quantitative indices also complement the waveform-based analysis and provide a reproducible basis for evaluating controller performance. It should be emphasized that these results characterize the experimentally investigated operating range and should not be interpreted as a proof of global performance for all possible operating conditions.

## 6. Conclusions

In the experimental studies conducted in the application setup, three tests were performed with the proposed controller under changes in input voltage, reference voltage, and load. The results were compared under the same operating conditions. The proposed controller maintains acceptable steady-state tracking accuracy within the investigated operating range, although a finite capacitor-voltage deviation is observed under severe load-step conditions. The results show that the proposed controller provides better dynamic performance. The proposed controller has +/−15 V steady-state error in the experimental studies and follows the reference voltage variations. In addition, since the proposed controller controls the input current on the DC side of the battery-assisted qZSI, its dynamic response is fast to input-voltage, reference-voltage, and load variations. Thus, the experimental results for step changes are satisfactory and consistent with the theoretical results.

## Figures and Tables

**Table 1:** Parameters used in the application and their values.

Definition and Parameter	Value
DC input voltage (*V_in_*)	140 V
V_C1_ capacitor voltage (*V_C_*_1_*)	170 V
Reference output voltage (v_o_*)	150 V
*C*_1_ = *C*_2_	450 V-1000 µF
*L*_1_ = *L*_2_	1.5 mH
*Cf* and *Lf*	20 µF ve 2 mH
Load resistance (*R_L_*)	15 Ω ve 30 Ω
PI gain values (K_p_ ve *K_i_*)	0.4–0.7 ve 30
PR gain values (*K_P_* ve *K_r_*)	0.0002 ve 2
Sampling period (T*_s_*)	100 µs
Switching frequency (*f_s_*)	10 kHz
Modulation (*m*)	0.7
Shoot-through mode duty ratio (*d*)	0.30

**Table 2:** Comparative summary obtained for different PI-controller gains.

Performance Metric	*K_p_* = 0.4, *K_i_* = 30	*K_p_* = 0.7, *K_i_* = 30	Comparative Assessment
Load resistance transition	30 Ω→15 Ω	30 Ω→15 Ω	Identical disturbance condition
Capacitor-voltage reference, *V_C_*_1_^∗^	170 V	170 V *	Same nominal reference
Capacitor-voltage deviation	≈15 V	Significantly reduced	Improved with *K_p_* = 0.7
Normalized *V_C_*_1_^∗^ deviation	≈8.8%	No significant steady-state deviation reported	Improved voltage regulation with *K_p_* = 0.7
Approximate transient duration	≈25 ms	≈20 ms	Quantitative value not reported for *K_p_* = 0.7
Battery-current response, *I_BAT_*	Approximately doubles after load change	Approximately 1 A → 2 A	Comparable response to increased load demand
Low-frequency battery-current ripple	Pronounced	Present	Further optimization may be required
AC output-voltage regulation	≈150 V, stable sinusoidal waveform	≈150 V, stable sinusoidal waveform	Satisfactory in both cases
Output-voltage THD	3.02%	3.68%	Lower THD with *K_p_* = 0.4
DC-side voltage regulation	Relatively poor	Improved	*K_p_* = 0.7 preferred
Overall performance	Lower THD but larger *V_C_*_1_ deviation	Improved DC-side regulation with acceptable THD	*K_p_* = 0.7, *K_i_* = 30 selected

* NR = Not Reported.

**Table 3:** Comparison of control and implementation characteristics of battery-assisted qZSI methods.

Criterion	Sun et al. [28]	Liu et al. [29]	Neves and Rolim [30]	Fan et al. [31]	Proposed
Application	PV and battery qZSI	PV and battery qZSI	Battery-assisted qZSI	Energy-stored qZSI	Battery-assisted qZSI
Operating mode	Grid connected	Grid connected	Grid connected	Battery-current control	**Standalone**
Main DC-side objective	PV and MPPT and power flow control	PV and MPPT and battery management	Battery-current control	Battery-current regulation	Battery and DC-side regulation
Main control approach	PI and feedforward + power control	Small-signal-based control	Power-based cascade control	State-space feedback	**Hybrid PI–PR**
Battery power management	Yes	Yes	Yes	Battery-current control	Yes
Small-signal/dynamic model	Limited system analysis	**Yes**	**Yes**	**Yes**	**Yes, revised model**
Experimental validation	**Yes**	**Yes**	No; simulation reported	NR	**Yes**
Simulation validation	Yes	Yes	**Yes**	NR	Yes/Experimental
Charge/discharge operation	**Experimentally investigated**	Battery management included	Battery-current regulation	Battery-current regulation	**To be added/revised**
Linear/nonlinear load tests	NR	NR	NR	NR	**Yes**
Input voltage disturbance test	NR	NR	NR	NR	**130 → 150 V**
Load-step test	NR	NR	NR	NR	**30 → 15 Ω**
Battery-current step test	Battery operation investigated	Battery-current dynamics investigated	Battery-current control	Battery-current control	**5 → 9 A**

NR = Not Reported.

**Table 4:** Quantitative dynamic performance comparison.

Performance Metric	[28]	[29]	[30]	[31]	Proposed
Voltage THD (%)	NR	NR	NR	NR	3.39
Current THD (%)	NR	NR	NR	NR	3.30
*V_C_*_1_ settling time(ms)	25	40	NR	NR	20
*V_C_*_1_ overshoot (%)	1.6	2	NR	NR	1.5
*V_C_*_1_ steady-state error (%)	3	5	NR	NR	3
*I_BAT_* settling time(ms)	30	25	25	20.6	20
*I_BAT_* overshoot (%)	3	2.5	2.2	2.1	1.5
*I_BAT_* steady-state error (%)	5	4.5	4.3	4.2	3

NR = Not Reported.

## Data Availability

The data presented in this study are available on request from the corresponding author. The data are not publicly available due to privacy.

## Abbreviations

The following abbreviations are used in this manuscript:

PWM	Pulse width modulation
ZSI	Impedance-source inverter
qZSI	Quasi-impedance-source inverter
DC	Direct current
AC	Alternative current
SMC	Sliding-mode control
SOC	Battery’s state of charge
PR	Proportional resonant
PI	Proportional integral control
SPWM	Sinusoidal pulse width modulation
THD	Total harmonic distortion
IGBT	Insulated Gate Bipolar Transistor
MPPT	Maximum Power Point Tracking
